# Assessment of Dietary Intake and Nutrient Gaps, and Development of Food-Based Recommendations, among Pregnant and Lactating Women in Zinder, Niger: An Optifood Linear Programming Analysis

**DOI:** 10.3390/nu11010072

**Published:** 2019-01-02

**Authors:** K. Ryan Wessells, Rebecca R. Young, Elaine L. Ferguson, Césaire T. Ouédraogo, M. Thierno Faye, Sonja Y. Hess

**Affiliations:** 1 Program in International and Community Nutrition, Department of Nutrition, University of California, Davis, CA 95616, USA; rryoung@ucdavis.edu (R.R.Y.); ctouedraogo@ucdavis.edu (C.T.O.); syhess@ucdavis.edu (S.Y.H.); 2 Department of Population Health, London School of Hygiene and Tropical Medicine, London WC1E 7HT, UK; elaine.ferguson@lshtm.ac.uk; 3 Helen Keller International, Niamey 0000, Niger; tfaye@hki.org

**Keywords:** linear programming, food-based recommendations, Optifood, micronutrient, deficiency, dietary intake, pregnant, lactation, women

## Abstract

Pregnant and lactating women in rural Niger are at high risk for inadequate intakes of multiple micronutrients. Thus, 24 h dietary recalls were conducted and analyzed for dietary intakes in this population (*n* = 202). Using linear programming analyses, micronutrient gaps in women’s diets were identified, food-based recommendations (FBR) to improve dietary micronutrient adequacy were developed, and various supplementation strategies were modelled. Energy intakes were below estimated requirements, and, for most micronutrients, >50% of women were at risk of inadequate intakes. Linear programming analyses indicated it would be difficult to select a diet that achieved recommended dietary allowances for all but three (vitamin B_6_, iron and zinc) of 11 modeled micronutrients. Consumption of one additional meal per day, and adherence to the selected FBR (daily consumption of dark green leafy vegetables, fermented milk, millet, pulses, and vitamin A fortified oil), would result in a low percentage of women at risk of inadequate intakes for eight modeled micronutrients (vitamin A, riboflavin, thiamin, B_6,_ folate, iron, zinc, and calcium). Because the promotion of realistic FBRs likely will not ensure that a low percentage of women are at risk of inadequate intakes for all modeled micronutrients, multiple micronutrient supplementation or provision of nutrient-dense foods should be prioritized.

## 1. Introduction

Maternal nutrition from the time of conception until two years post-partum, a period known as the first 1000 days, is critical for maternal and child health [1]. Undernutrition during pregnancy is a risk factor for maternal mortality and fetal growth restriction, which increases the risk of neonatal deaths and contributes to impaired post-natal linear growth and development [1]. Undernutrition during lactation adversely affects the concentrations of some macro- and micronutrients in breastmilk, which may negatively impact infant morbidity and mortality [2]. In low-income countries, it is particularly challenging for women to meet their macro- and micronutrient requirements during pregnancy and lactation [3]. In Niger, the lifetime risk of maternal death is 1 in 23 women and 11.4% of children die before reaching 5 years of age [4]. In a recent cross-sectional survey conducted among pregnant women in Zinder, Niger, 27% of pregnant women had inadequate gestational weight gain and 25% had low mid-upper arm circumference, indicative of undernutrition [5]. In addition, the prevalence of multiple micronutrient deficiencies was indicative of a severe public health problem. 45% of pregnant women were deficient in > 3 micronutrients (iron, zinc, vitamin A, folate, vitamin B_12_), 79% were anemic, and less than 20% had adequate minimum dietary diversity [6]. Although information on the nutritional status of lactating women is limited, increased physiological requirements, frequent reproductive cycling and resource constraints make undernutrition likely. Overall, the Zinder region of Niger is considered a high risk livelihood zone, subject to severe food access constraints due in part to variable rainfall and frequent droughts, imbalanced agro-pastoralism, and high poverty levels. The 2011 National Survey on Living Conditions: Household and Agriculture in Niger (ECVMA) estimated that 47.7% of the population in the Zinder Region was living below the poverty line [7]. Chronic moderate and severe food insecurity affected 27.2% and 12.5% of the population in the Zinder region of Niger, respectively. During the lean season, these prevalences increased to 51.2% and 32.7%, respectively [7]. 

Evidence-based approaches to improve nutritional status among pregnant and lactating women include supplementation (e.g., iron and folic acid, multiple micronutrients, and balanced protein and energy supplementation), food fortification (e.g., mass fortification of cereals, oils, and condiments), and dietary counseling to promote the consumption of nutritionally dense foods [8,9,10,11,12]. Software tools for decision-making in nutrition programs have been developed to support advocacy and decision-making (e.g., Lives Saved Tool, LiST; Cost of the diet, COD), plan and optimize interventions (e.g., Intake Modeling, Assessment and Planning Program, IMAPP; Optifood), and optimize cost-benefits of combined interventions (e.g., MINIMOD) [13]. Optifood is based on the mathematical technique of linear programming, and was recently developed by the London School of Hygiene & Tropical Medicine, the World Health Organization (WHO) and USAID Food and Nutrition Technical Assistance Project (FANTA)/FHI 360. Optifood allows users to identify population-specific dietary nutrient gaps and develop food-based recommendations (FBR) centered on locally available and acceptable foods, accounting for existing dietary patterns and economic feasibility [14]. In addition, the approach can be used to evaluate the ability of existing and novel nutrient supplements and fortified foods to meet population-specific nutritional requirements [15,16,17,18,19], although it has, to date, primarily been used for infants and young children.

The primary objectives of the present study were to (1) assess dietary intake and nutritional adequacy among pregnant and lactating women in the Zinder region of Niger, (2) develop FBR for nutritional counseling accounting for current consumption patterns and the local availability of affordable nutrient-rich foods, and (3) identify any shortfalls requiring nutrient supplementation or fortification. The present study illustrates how the Optifood tool can be used to inform program and policy decisions regarding strategies to improve dietary adequacy among pregnant and lactating women in food insecure populations.

## 2. Materials and Methods 

### 2.1. Study Design and Participants

The present assessment of dietary intake and daily nutrient intakes among pregnant and lactating women is a cross-sectional study embedded into the Niger Maternal Nutrition (NiMaNu) Project. The NiMaNu project was a program-based effectiveness trial in the Zinder region of Niger, designed to assist the Nigerien Ministry of Public Health in its efforts to improve the nutritional and health status of pregnant women through multiple strategies to increase antenatal care attendance and adherence to iron folic acid (IFA) supplementation. The overall NiMaNu programmatic intervention, study design and data collection methods have been reported in detail elsewhere [6,20,21]. Briefly, 18 governmental integrated health centers located in two districts (Zinder and Mirriah) of the Zinder Region of Niger were randomly assigned to time of enrollment in the NiMaNu project from March 2014 – September 2015, and 2307 pregnant women in surrounding rural villages participated in the study. The present dietary intake assessment survey was implemented from May – October 2015, exclusive of the month of Ramadan and included women from nine villages within the catchment areas of three integrated health centers enrolled in the baseline NiMaNu survey during that time frame. 

Pregnant and lactating women were identified using the random walk method [22] and were eligible to participate in the dietary intake assessment survey if they lived in the catchment area of the NiMaNu project, were in their second or third trimester of pregnancy or breastfeeding an infant or young child < 23 months of age from a singleton birth, were > 19 years of age, and provided written informed consent. Women were ineligible to participate if they were unable to provide consent due to impaired decision-making ability, or if they (or their breastfeeding child) had an illness warranting immediate hospital referral or a chronic or congenital illness interfering with dietary intake, as assessed via a structured questionnaire and the professional judgement of the fieldworkers and study coordinator (government certified midwives and medical doctor, respectively). 

### 2.2. Ethical Considerations

The NiMaNu Project was approved by the National Ethical Committee in Niamey (Niger) (005/2013/CCNE; 007/MSP/CCNE/2015) and the Institutional Review Board of the University of California, Davis (USA) (447971). Consent materials were presented in both written and oral format, in the presence of a neutral witness. Informed consent was documented with a written signature or a fingerprint prior to enrollment in the study. The study was registered at www.clinicaltrials.gov as NCT01832688. 

### 2.3. Data Collection

#### 2.3.1. Socio-Demographic Characteristics and Anthropometry

Information on socio-economic and demographic characteristics of the woman and her household, pregnancy and health status, food security, and knowledge, attitudes and practices pertaining to antenatal care and nutrition were collected via structured interviews by trained female fieldworkers [6,20,21]. The survey on knowledge, attitudes and practices included questions of knowledge (e.g., benefits of IFA and recommended foods to consume during pregnancy and lactation), attitudes (e.g., perceived importance of IFA, reasons for compliance or non-compliance with IFA supplementation, and identification of foods they would like to consume in greater quantity during pregnancy and lactation), and practices (e.g., IFA consumption, changes to physical labor and dietary intakes during pregnancy and lactation). Household food insecurity was assessed using the Household Food Insecurity Access Scale (HFIAS) of the Food and Nutrition Technical Assistance/USAID [23]. Height, weight, mid-upper arm circumference (MUAC) and symphysis-fundal height were measured by trained and standardized anthropometrists. Lightly clothed women were weighed to 50 g precision (SECA 874, Seca, Hamburg, Germany) in duplicate. Women’s height (SECA 213, Seca, Hamburg, Germany), MUAC (ShorrTape© Measuring Tape, Weigh and Measure, Olney, MD, USA) and, among pregnant women, symphysis-fundal height (ShorrTape© Measuring Tape, Weigh and Measure, Olney, MD, USA) were measured in duplicate to 0.1 cm precision. If the two measurements were >0.2 kg (weight) or >0.5 cm apart (height, MUAC, and symphysis-fundal height), a third measurement was taken and the mean of the two closest measurements was calculated. Undernutrition was defined as a MUAC < 23 cm among pregnant women or a BMI < 18.5 kg/m^2^ among lactating women, respectively [24]. Gestational age was estimated as a weighted average of the following obtained information: reported last menstrual period, time elapsed since quickening, and two fundal height measurements taken approximately one month apart [20,25].

#### 2.3.2. 24 h Recall Data Collection

Three trained fieldworkers administered interactive quantitative 24 h dietary recall interviews, following the multi-pass approach developed for use in rural populations in low-income countries with low rates of literacy; study-specific data collection forms are available on the Open Science Framework platform [20,26]. Single 24 h dietary recalls were conducted in all participants; duplicate 24 h dietary recalls were attempted in a sub-sample of women (20%). 24 h dietary recalls were proportionally collected on weekdays (Monday-Thursday) and weekends (Friday-Sunday). Participating women were visited twice in their homes, two days apart. At the first visit, two days prior to the scheduled 24 h dietary recall, the purpose of the dietary interview was explained and participants were given a pictorial chart of common foods and a cup and bowl to use for their individual portions on the following day, to stimulate recall and allow the women to more accurately estimate portion sizes. On the day of the 24 h dietary recall interview, fieldworkers used neutral probing questions to help participants recall all the foods and drinks they had consumed during the preceding 24 h period (pass one). After this list was obtained, fieldworkers probed for more specific descriptions of all items listed in the first pass (pass two; e.g., brand names, recipes, cooking methods, waste or non-consumed parts, etc.). In the third pass, portion sizes were estimated using an electronic scale (when commonly consumed foods and ingredients carried by fieldworkers or foods or ingredients still available in the household could be directly weighed), equivalent volumes of water or dry good consumed (e.g., dry beans, dry couscous), pre-calibrated local utensils, rulers, modeling clay or monetary value. Finally, the fourth pass was used to review the recall and ensure all items were included and recorded correctly. A dietary diversity score (minimum dietary diversity for women; MDD-W) was calculated using 24 h recall data [27]. In addition, for all food items reported on the 24 h recall, women were asked to report the number of times per week or month that each food was typically consumed. For recipes or mixed dishes that were prepared by the index woman, information was collected on the type and amounts of ingredients, cooking methods, the total amount of recipe prepared, and the proportion she consumed. For composite dishes not prepared by the respondent and for staple foods with little intra-recipe variability, general recipes were constructed by commissioning three local women to prepare each recipe, or by compiling individual recipes obtained from women enrolled in the dietary intake assessment survey. In all cases, weights of raw ingredients were averaged in proportion to the total amount of recipe prepared. For all measurements not directly expressed in grams of food consumed, conversion factors were collected by fieldworkers in triplicate (e.g., cost data from local markets, densities of specific foods to convert estimated volumes of water or grams of dry goods to grams of prepared food, etc.), and applied to collected data, such that all data were ultimately expressed in grams of food consumed. In addition, all data were expressed in total cost of food consumed, using fieldworker acquired cost data from local markets, market survey data (see below) and participant-reported data. 

#### 2.3.3. Market Survey

Market surveys were conducted over seventeen months in all primary markets of the NiMaNu study area. From May 2014 – September 2015, these markets (*n* = 10) were surveyed a total of 55 times (Zinder regional market, *n* = 26; 9 local markets in the Mirriah district, *n* = 29). Information was collected on food availability and cost, using a structured questionnaire based on the National Survey of Household Budget and Consumption and pre-tested in local markets [28]. Data are expressed as cost per 100 g edible portion. 

#### 2.3.4. Food Composition Table

Food nutrient values were obtained from the Optifood internal reference food composition table, which contains nutrient composition data for approximately 2000 foods. Nutrient values for any food items not contained in the Optifood food composition table were compiled from the INFOODS Regional Nutrient Database for West Africa, the WorldFood System International Mini-list, and the United States Department of Agriculture Nutrient Database for Standard Reference, Release 28 (USDA SR28) [29]. The phytate contents of foods, including adjustments for fermentation, were imputed from study-specific analyses of food samples, or where data were not available, from the IML or a database compiled by Wessells et al. [30]. Nutrient contents of raw foods in the food composition table, when consumed in the cooked state, were adjusted for nutrient losses during cooking using retention factors from the USDA [31]. Refuse factors, obtained from the aforementioned databases, were used to convert all reported food units to edible portions. 

Nutritional information from commercially available foods was obtained to account for fortification. Vegetable oils were assumed to be fortified with retinyl palmitate at the minimum at-market concentration of 11 mg/kg, as established by the West African Economic and Monetary Union (UEMOA) standards [32]. Wheat flour was assumed to be fortified with iron (60 mg/kg) and folic acid (2.5 mg/kg) [33,34]. Maggi brand bouillon cubes were assumed to be fortified with iron at a concentration of 600 mg/kg (based on package labelling and independent laboratory analyses) [35]; for the present Optifood analyses, all bouillon cubes were considered fortified. The nutrient composition of supplements (IFA, UNICEF/WHO/UNU international multiple micronutrient preparation (UNIMMAP)) and supplemental food products (Supercereal (i.e., Corn Soy Blend Plus, CSB+), Small Quantity Lipid-Based Nutrient Supplements for pregnant and lactation women (SQ-LNS P&L), Plumpy’Mum) was obtained from manufacturers specifications; details are in Table S1. 

#### 2.3.5. Dietary Reference Intakes

The dietary reference intakes (DRI) of the Food and Nutrition Board (FNB) of the Institute of Medicine (IOM; National Academies, USA) were used for these analyses (Table S2) [36,37,38,39,40]. Estimated energy requirements (EER) were calculated using IOM predictive equations by physiological status (specific to 2nd or 3rd trimester among pregnant women, and 0–6 or >6 months post-partum among lactating women) and physical activity level (PAL; assumed active PAL 1.27). The acceptable macronutrient distribution ranges (AMDR) of dietary protein and fat were considered to be 10–35% and 20–35% of energy, respectively. Nutritional adequacy of eleven micronutrients was assessed (thiamin, riboflavin, niacin, folate, vitamins B_6_, B_12_, A and C and iron, zinc and calcium). The estimated average requirement (EAR) and recommended dietary allowance (RDA) for micronutrients were IOM DRI recommendations, with the exception of the iron bioavailability among lactating women and the calcium recommendations. Specifically, for lactating women, the fractional absorption of iron was assumed to be 10%, due to lower bioavailability which may be encountered in predominantly vegetarian diets with limited diversity; thus increasing recommendations above those set by the IOM, yet still in line with recommendations from the WHO [3,41]. DRI for pregnant women were not adjusted, due to increases in iron absorption during later pregnancy [42]. In addition, models were run using both the calcium recommendations set by the IOM, as well as those recommended by WHO/FAO for settings where animal source foods provide less than 20–40 g/day of protein [41].

### 2.4. Data Analyses

Data were entered using EpiData version 3.1 (EpiData Association, Odense, Denmark). Dietary data were prepared in RedCap and SAS System software for Windows release 9.4 (SAS Institute, Cary, NC, USA). Statistical analyses were completed with SAS System software for Windows release 9.4 (SAS Institute, Cary, NC, USA). Descriptive statistics were calculated for all variables. The distribution of usual micronutrient intakes and daily per capita reported cost of foods consumed was estimated using the National Cancer Institute (NCI) method to adjust for intra-individual variation in dietary intake; the EAR cut-point method was applied to estimate the prevalence of inadequate intake [43,44]. Differences in market availability and median prices of specific foods by season were analyzed using logistic regression with Firth’s adjustment and the Kruskal Wallis test on ranked data, respectively. Data are presented as mean ± SD for normally distributed variables (Shapiro-Wilk statistic, W > 0.97), and the median and IQR for non-normal values. The alpha value is 0.05. 

### 2.5. Optifood Analyses

#### 2.5.1. Preparation of Linear Programming Model Parameters

Summary statistics from the dietary intake assessment survey (24 h recalls), including a list of foods consumed, food serving sizes and food patterns, were used to define the linear programming model parameters. The list of foods consumed included those consumed by > 5% of each target group and nutrient-dense foods consumed by < 5% of the target group, but with the potential to be promoted for consumption. It excluded all non-nutritive foods. Food serving sizes were defined as the median serving size (grams/meal) among consumers of each food; for staple foods (i.e., “grains and grain products”), food serving sizes were defined as 75th percentile, to allow for adequate flexibility to modeled energy. The minimum and maximum number of meals per week that a food could be consumed was calculated using data from the food frequency questionnaires. Minimum frequency was defined as 0 servings/week; maximum frequency was defined as the 90th percentile of the food frequency distribution for each group (pregnant and lactating women), with a lower limit of 1 serving/week and an upper limit of 21 servings/week. Weekly food consumption patterns for specific food groups and food sub-groups were included in the model (minimum and 90th percentile) to ensure that the diets modeled conformed to the range of food patterns observed in the target group. Estimated median servings per week, for each food group, defined food group goals in one of the Module II goal programming models. If the median value was zero, then a value of 0.1 servings per week was entered to avoid division by zero. These aforementioned parameters were used to define the model constraint levels used in the linear programming models analyzed in the WHO Optifood Software (version 4.0.14.0). 

#### 2.5.2. Development of Modelled Diets

The Optifood linear programming software (Modules I to III) was used to check model parameters, identify problem nutrients in the current dietary patterns of the target groups and to develop and test population-specific FBR for pregnant and lactating women [14,45]. Nutritionally “best” diets for the target population were generated based on established constraints and goals to achieve or exceed nutrient requirements (one model) or to achieve median food group patterns and achieve or exceed nutrient requirements (another model). Results from these goal programming models identified nutritious food sources, problem nutrients and alternative food-based recommendations to test. Next, linear programming was used, in Module III, to compare alternative sets of these food-based recommendations for nutritional adequacy and cost. Independent models for each micronutrient simulated the minimized (worst-case scenario) and maximized (best-case scenario) values of the nutrient intake distribution and provided cost estimates for each scenario [45].

Two series of analyses were done using the Optifood software tool. In the first series of analyses, the model energy constraint was equal to the reported mean energy intake for each target group (1812 kcal/day and 2280 kcal/day for pregnant and lactating women, respectively), henceforth referred to as “reported diet” models. A second series of diets was then modeled, in which the model energy constraint was increased to approximate the provision of an “added meal” (~600 kcal) per day [46] among pregnant women (2415 kcal/day), or to match estimated energy requirements in lactating women (2622 kcal/day). Subsequently, series of linear programming models were run to test alternative intervention products (Table 1). 

“Problem” nutrients were defined as nutrients where the nutrient did not achieve 100% of the RDA in the maximized best-case scenario; these are nutrients that will likely remain inadequate in the population given the local food supply and food patterns, even if women were to follow the FBR. Dietary adequacy for each nutrient was defined as the worst-case scenario for that nutrient being > 65% of the RDA. A worst-case-scenario level ≥ 65% indicates, if women achieve the FBR, then a low percentage of the population would be at risk of inadequate intakes for that nutrient. 

### 2.6. Sample Size

Sample size estimates for the collection of dietary data among pregnant and lactating women were based on sample sizes previously reported in the literature [15,47,48]. Based on this information, and including an attrition rate of 10%, it was planned to enroll 110 pregnant women concurrent to their participation in the NiMaNu study and 110 lactating women residing in the same catchment areas. Duplicate non-consecutive day 24 h recalls were attempted in 20% of enrolled women within seven days, in order to examine intra-individual variation in nutrient intake. 

Due to study resources and logistics, it was not always possible to implement the NiMaNu study and dietary intake assessment survey simultaneously as planned. Thus, only 56 pregnant women participating in the NiMaNu study were also enrolled in the present dietary assessment survey; an additional 48 pregnant women were enrolled in the dietary intake assessment survey only or subsequent to their completion of the NiMaNu study. In all cases, recruitment procedures were the same and data for the dietary intake assessment survey were collected using identical protocols and fieldworkers. In addition, an oversampling of lactating women occurred in the first village due to a miscommunication with fieldworkers. Thus, a post-hoc random sample of enrolled lactating women from the first village who completed the 24 h dietary recall was included in the analyses (*n* = 20 of 41).

## 3. Results

**Figure 1:**
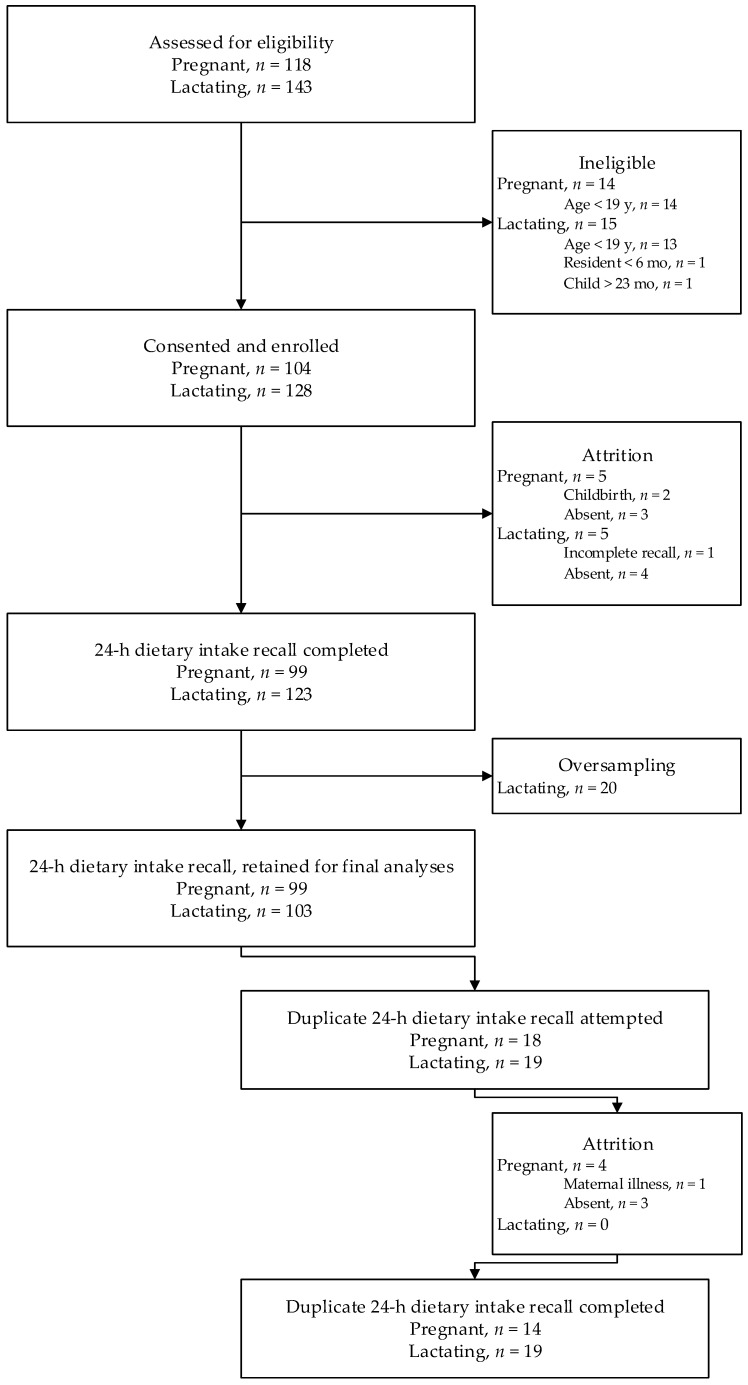
Flowchart of participant progression through the dietary intake assessment survey.

A total sample of 202 participants (103 lactating women and 99 pregnant women) was retained for analyses (Figure 1). The survey was primarily conducted during the “lean” season, and the majority of women reported moderate or severe household food insecurity. Only 16% of women reported adequate dietary diversity and >20% of participants were undernourished (Table 2).

### 3.1. Usual Dietary Intakes

Energy intakes in pregnant and lactating women were substantially below EER, and pregnant women reported consuming significantly fewer calories than lactating women, despite similar EER (*p* < 0.0001) (Table 3). Among pregnant women, reported mean energy intake did not differ by trimester (*p* = 0.548). Median percent contribution of energy from carbohydrates (70%) was slightly above the upper limit of the AMDR, and those of protein and fat were at or slightly below the lower limit of the acceptable macronutrient distribution ranges (10% and 20%, respectively). Usual dietary intakes of vitamin A, thiamin, riboflavin, niacin, folate and vitamin C were inadequate among >50% of pregnant and lactating women; usual dietary calcium and vitamin B_12_ intakes were inadequate for all women. Median (IQR) daily per capita reported cost of foods consumed was 0.35 € (0.28, 0.45) and 0.39 € (0.30, 0.49) for pregnant and lactating women, respectively.

### 3.2. Optifood

#### 3.2.1. Dietary Patterns and Linear Programming Model Parameters

Table S3 shows the foods commonly consumed by pregnant and lactating women, the median serving sizes among consumers (75th percentile for staple foods), and the maximum frequency of consumption (upper constraint of servings/week), both as the 90th percentile of reported intake and as adjusted to allow for an “added meal” per day in the Optifood models. The median and maximum number of servings per week by food group, and food sub-group, are presented in Table S4. A total of 66 individual food items were reported as consumed by pregnant and/or lactating women. Of these, 30 and 34 of these foods were consumed by ≥5% of lactating and pregnant women, respectively; and FBR generated by Optifood modeling were restricted to these commonly consumed foods. Grains and grain products, dark green leafy vegetables (DGLV), vegetable oils and legumes (pulses) were principle components of the diet. Food items recorded as being available in at least 11 of the 55 market surveys completed are presented in Table S5. In general, there were limited seasonal differences in availability and median prices. Of note, starchy roots, animal source foods, particularly meat, fish and eggs, and fruits were available in markets, but were rarely consumed by pregnant and lactating women (cumulatively <0.5% of eating occasions), and median serving sizes among consumers were small (e.g., meat, 34 g and egg, 64 g). Median prices per 100 g edible portions of animal source foods, excluding dairy, ranged from 0.22–0.47 €, compared to 0.03–0.10 € for grains and grain products.

#### 3.2.2. Linear Programming

##### Reported Diet and Food-based Recommendations

Based on reported dietary intakes among pregnant and lactating women, the nutritionally “best” diets, with and without adherence to dietary food patterns (Module II), indicated that only zinc and iron reached >100% of the RDA (Tables S6 and S7). Linear programming analyses indicated that it was difficult to select a diet that achieved RDAs for all but three (vitamin B_6_, iron and zinc) of the modeled micronutrients (Module III maximized diets where the best-case scenario is > 100% of the RDA; Table 4 and Table 5). The remaining eight micronutrients that were modeled were identified as “problem” nutrients and likely to remain inadequate among this population, given the local food supply and food patterns. Thus, model constraints were changed prior to selecting (Module II) and testing (Module III) FBRs for these women (see below). Among pregnant women, including daily iron and folic acid supplements as the standard-of-care practice would ensure a low percentage of women would be at risk of inadequate intakes of folate, in addition to the already adequate intakes of iron, vitamin B_6_ and zinc, but would do nothing to alleviate the remaining micronutrient inadequacies.

##### “Added Meal” and Food-Based Recommendations

Since observed mean energy intakes were substantially less than EER, scenarios were modeled which included the provision of an “added meal” per day (Table 1 and Table S3). In Module II, the nutritionally “best” diet without considering current food patterns achieved 100% of the RDA for seven micronutrients among pregnant women and six micronutrients among lactating women, indicating at least one modelled diet can achieve RDAs for these nutrients (Tables S6 and S7). Final sets of FBR were selected (Table 6), with and without IFA supplements for pregnant women, which resulted in worst-case scenario values >65% of the RDA for thiamin, riboflavin, B_6_, folate, iron, zinc and calcium for both pregnant and lactating women, plus vitamin A for pregnant women only (Table 4 and Table 5). However, even if the set of FBR was successfully adopted, vitamins B_12_, C and niacin values were < 65% of the RDA indicating the FBRs would not ensure a low percentage of pregnant and lactating women would be at risk of inadequate intakes for these nutrients. In addition, vitamin A values were < 65% of the RDA in lactating women, due to higher dietary recommendations. The minimum cost of a diet including an added meal per day, combined with FBR, was estimated to be 0.43 €/day for both pregnant and lactating women. 

When rarely consumed nutrient-dense animal source foods were included in the models along with the aforementioned FBR (e.g., one egg/day or 100 g meat/day), no model was able to ensure women were at low risk of inadequate intakes for all micronutrients; vitamins B_12_, C, and niacin remained <65% of the RDA in one or more models (Table S8). In addition, the estimated cost of the diet increased by 35–59% in comparison to modeled diets providing one added meal per day and including the best set of FBR.

##### Intervention Products and Food-based Recommendations

FBRs with the provision of an added meal, with or without IFA for pregnant women or the inclusion of a rarely consumed nutrient-dense food did not ensure that a low percentage of pregnant or lactating women were at risk of inadequate intakes for all micronutrients, therefore additional supplementation strategies were modeled (Table 1). Consuming one added meal per day, plus a UNIMMAP supplement or a food-based product (SQ-LNS P&L, Plumpy’Mum and Supercereal, would ensure that a low percentage of pregnant and lactating women would be at low risk of inadequate intakes for almost all modeled micronutrients with the consistent exception of calcium (Table 4 and Table 5). Specific sets of FBR to complement each intervention product focused on calcium-rich food sources, including DGLV, fermented milk, pulses and/or millet (Table 6). When lower calcium recommendations were used (775–800 mg/day vs. 1000 mg/day), the provision of Plumpy’Mum reduced the need for fermented milk to be included in FBR, and the provision of SQ-LNS eliminated it (Tables S9–S11). Including intervention products, with FBR in the modeled diets, decreased the estimated cost of the diets, as models did not account for costs associated with the products themselves or programmatic implementation (Table 4 and Table 5).

## 4. Discussion

The present analyses indicated that energy intakes in pregnant and lactating women in rural Zinder, Niger were low compared to their estimated energy requirements, which is corroborated by the finding of a high prevalence of undernutrition in the population. Additionally, analysis of usual dietary intakes indicated that the prevalence of inadequate micronutrient intake was greater than 50% for eight and nine of the eleven micronutrients evaluated, among lactating and pregnant women respectively. Similarly, a 2010 systematic review reporting micronutrient intake among women in resource-poor settings concluded that inadequate intakes of multiple micronutrients were common, with reported mean or median intakes in over 50% of the studies below the EAR [3]. In addition, these present findings are supported by recently published data on biochemical micronutrient status among pregnant women in the same population, which indicated that approximately 25–50% of women had low plasma concentrations of zinc, folate and vitamin B_12_, and iron deficiency, and >75% had marginal vitamin A status and anemia [6]. 

Only one in six women reported adequate dietary diversity, consuming at least five of ten defined food groups the previous day and night. Initial Optifood analyses, based on reported diets, revealed it would be difficult to meet nutrient recommendations given current dietary patterns, unless energy intakes were increased. A second series of analyses, based on the recommendation to include one added meal per day, indicated that an increased caloric intake plus FBR to increase the weekly consumption of nutrient-dense foods (e.g., DGLV, dairy and legumes), could improve the nutritional quality of the diet. However, even if women adhered to the best set of FBR modeled in these analyses, FBR alone, with or without IFA supplements included as standard antenatal care, could not ensure that a low percentage of pregnant or lactating women would be at low risk of inadequate intake for all 11 micronutrients modeled. Dietary adequacy could not be ensured for vitamins A, C, B_12_ and niacin. In general, nutrient-dense foods were either not commonly consumed (i.e., meat, fish and eggs, vitamin C-rich fruits and vegetables) and thus did not appear as options for inclusion in the FBR, or were consumed in insufficient quantities to meet dietary recommendations (vitamin A-rich DGLV and fortified vegetable oils). 

Numerous linear programming analyses in other low-income settings have indicated the potential of FBR and locally available foods to improve dietary micronutrient adequacy among the infants and young children although in most settings combinations of FBR with fortified foods or dietary supplements would be required [15,16,18,47,50]. Among women of reproductive age in low income countries, linear programming analyses have indicated that in some instances micronutrient adequacy may be achievable with FBRs, when nutrient-dense foods are included that are typically infrequently consumed, (e.g., animal source foods such as liver and small fish) [42,49,51]. Similar to our findings, other studies have concluded that a modest set of FBR, in combination with micronutrient or food-based supplements, would be necessary to meet nutrient adequacy among women of reproductive age, or might be more feasible and acceptable than FBR alone [42,47].

The present study is a modeling analysis based on reported dietary intakes, and does not present results from an efficacy or effectiveness trial. Thus, it is not possible to make strong conclusions about the feasibility of different proposed solutions in the present study. For example, when modeling IFA supplements as a dietary supplement, 100% coverage and full adherence was assumed. However, in reality the coverage and the adherence are much lower. Among pregnant women enrolled in the baseline survey of the NiMaNu Project (*n* = 923), only 44% had received IFA supplements during their current pregnancy, and 69% of these women reported adherence to IFA supplementation as recommended (i.e., consumed IFA daily in the previous week) [21]. Nevertheless, this research does highlight the critical necessity of additional interventions in this population, and provides general guidelines for proposed solutions. FBR without monetary support, rations or the inclusion of supplements, would require large changes to current dietary patterns (increased caloric consumption particularly among pregnant women, consumption of nutrient-dense foods including dairy and animal-protein foods, etc.) that may not be feasible, considering household resource constraints and food consumptions patterns during pregnancy and lactation. Thus, the recommendation to include one additional meal per day, as recommended in the Essential Nutrition Actions [46], in addition to a set of FBR, may be difficult in this context, particularly for pregnant women. 

In the present study, >50% of women were moderately to severely food insecure, and 68% were below the national poverty line, based on daily food consumption expenditures alone (0.44 €) [52]. Therefore, increasing the median daily cost of the diet from 0.37 € (0.30, 0.49) to a projected *minimum* cost of 0.43 €/day, may be cost-prohibitive for the majority of women, where food expenditures are already accounting for >50% of total expenditures and the consumption aggregate poverty line (food and non-food expenditures) is 0.67 €/day [52]. The Prospective Urban Rural Epidemiology Study examined availability and affordability of fruits and vegetables in 18 countries, and reported that households in low-income countries (Bangladesh, India, Pakistan and Zimbabwe) would have to spend >50% of their household income to purchase two servings of fruits and three servings of vegetables per individual per day (compared to 2% of household income in high-income countries) [53]. Market surveys indicated that multiple nutrient-dense foods including meat, fish and eggs, fruits and vitamin A- and C-rich vegetables were available in local and regional markets, irrespective of season. However, these foods were not commonly consumed, as reported on 24 h dietary recalls. Thus, although availability and access, particularly at the village level, may be barriers to consumption, decisions regarding dietary intake may also be driven by cost or food preferences based on taste, convenience and storage capabilities [42]. In a recent longitudinal qualitative study to assess the determinants of dietary practices during pregnancy in a neighboring region of rural Niger, pregnant women noted physiological, household, community and structural level constraints to consuming their ideal pregnancy diets (e.g., maternal morbidity and food aversions, limited financial autonomy of women regarding household food purchasing, limited supply of preferred food items in local markets and systemic poverty) [54]. 

Combining the provision of balanced protein-energy supplementation with food-based recommendations may ensure that a low percentage of women would be at risk of inadequate nutrient intakes, with projected minimum per capita diet costs of 0.19–0.32 €/day. However, even adherence to FBR combined with supplementation products may be difficult for women to achieve. For example, most FBR relied heavily on the consumption of fermented milk (7–14 servings/week; equivalent to ~100–200 mL per day) to meet calcium requirements. Adhering to these FBR may be challenging, as currently, only 25% of pregnant and lactating women reported consuming fermented milk in their 24 h dietary recalls, and 90% reported consuming <7 servings/week. Alternative interventions include the provision of comprehensive “food baskets”, containing both commodities and nutrient-dense foods, cash transfers, for pregnant and lactating women to purchase nutrient-dense foods at local markets, or homestead food production. However, these scenarios do not include any production or distribution costs for the supplemental products, food baskets or cash transfers and assume that all costs would be borne by the governmental or non-governmental organizations rather than the target population. In all proposed solutions, well designed and implemented behavior change interventions would be necessary to evaluate adherence to food-based recommendations and supplementation among pregnant and lactating women and further research would be necessary to evaluate feasibility and acceptability. 

Optifood models rely on assumptions regarding dietary requirements and the accuracy of nutrient composition data. Previous research has shown that varying assumptions regarding nutrient requirements, bioavailability and absorption, and the nutrient composition of foods affects estimated global prevalences of inadequate micronutrient intake [30]. Of note, iron and zinc were not identified as problem nutrients in the present study, despite prevalences of iron deficiency (low ferritin and high soluble transferrin receptor) and low plasma zinc concentrations ranging from 21–41% among pregnant women in the same study area [6]. Given the documented deficiencies in these micronutrients through biochemical assessments, it may be prudent to interpret the modeling results with caution. Recommended dietary allowances for iron and zinc in pregnant and lactating women were based on those established by the FNB/IOM; iron RDA assumed 25% and 10% absorption, respectively, and zinc RDA assumed 27% and 38% absorption, respectively [3,37]. Given the predominantly plant-based diets of the study participants, variable fermentation practices and limited data on the phytate content of common foods, it is possible that iron and zinc bioavailability, and thus absorption, were lower than estimated, thus increasing dietary intake requirements. Finally, these analyses do not account for the conversion of tryptophan to niacin, which should be taken into account in the context of purported inadequate niacin intakes. However, in spite of all these uncertainties in specific micronutrient recommendations, linear programming of various scenarios consistently indicated that overall, pregnant and lactating women in this population have difficulty meeting nutrient recommendations, given locally available foods and food patterns.

The present study had several strengths and weaknesses. Estimations of usual intakes and development of FBR through linear programming rely on the quality of the dietary data collected. This study used quantitative 24 h dietary recall data, which is subject to omissions, under- or- over-reporting of consumption and inaccuracies in portion size estimates. To minimize errors and bias, an interactive, systematic multi-pass method, developed specifically for use in rural populations in low-income countries with low rates of literacy, was used [26]. Certified midwives were hired as fieldworkers and were rigorously trained and supervised in data collection. Prior to the recall day, participants received training in portion size estimation, standard dishware and pictorial memory-aide charts. In addition, numerous methods were employed to estimate portion size, including pre-prepared staple foods, standard dishware and household utensils and purchase price of foods bought outside the home. In spite of these precautions, it is likely that recalls were subject to inaccuracies in reporting. In addition, estimates also depend on the accuracy and validity of food composition tables. Nutrient composition data are limited for locally available and wild food items, and it is difficult to account for the retention and bioavailability of nutrients in home-processed foods (e.g., milled, fermented, dried, cooked, etc.). Models assumed universal fortification of vegetable oils and wheat flour per national policy; however, the true extent of coverage is unknown. In addition, all bouillon cubes were assumed to be fortified with iron, but fortification is voluntary and not practiced by all producers. If vegetable oils, wheat flour or bouillon cubes consumed by this population are unfortified, or micronutrient retention in fortified products is low, vitamin A, iron and folate may be more likely to be problem nutrients than it appeared in these analyses. Finally, these analyses were limited to the micronutrients for which there are reference data available in the Optifood software; analysis of the micronutrient adequacy of additional micronutrients would be of interest.

## 5. Conclusions

In summary, linear programming of various scenarios consistently indicated that overall, pregnant and lactating women in this population have difficulty meeting nutrient recommendations given locally available foods and dietary patterns. Providing IFA supplements to pregnant women as the current standard of care in this population is inadequate to address multi-micronutrient inadequacies. In addition, modeling possible FBR suggests that these would not adequately address micronutrient deficiencies and may be cost-prohibitive for the local context. Thus, multiple micronutrient supplementation, and the provision of nutrient-dense food-based interventions should be considered. Effectiveness trials and program implementation research will be instrumental to determine the likelihood of various scenarios (sets of FBR and multiple micronutrient or balanced protein-energy supplements) to improve micronutrient intakes and biochemical and functional nutritional status.

## Figures and Tables

**Table 1:** Series of linear programming models developed, with alternative intervention products, using the Optifood software tool ^1^.

	Pregnant Women		Lactating Women	
	Model Energy Constraint ^2^ (kcal/day)	Modeled Intervention Product (per day)	Model Energy Constraint ^2^ (kcal/day)	Modeled Intervention Product (per day)
Reported diet	1811.9	---	2279.5	---
Reported diet + IFA (standard of care)	1811.9	1 IFA	---	---
Added meal diet	2414.8	---	2622.0	---
Added meal diet + IFA	2414.8	1 IFA	---	---
Added meal diet + UNIMMAP ^3^	2418.8	1 UNIMMAP	2622.0	1 UNIMMAP
Added meal diet + Supercereal (CSB+) ^3,4^	2418.8	1 serving of CSB+ (500 kcal)	2622.0	1 serving of CSB+ (500 kcal)
Added meal diet + SQ-LNS (P&L) ^3^	2418.8	1 serving of SQ-LNS (118 kcal)	2622.0	1 serving of SQ-LNS (118 kcal)
Added meal diet + Plumpy’Mum ^3^	2418.8	1 serving of Plumpy’Mum (515 kcal/day)	2622.0	1 serving of Plumpy’Mum (515 kcal/day)

^1^ CSB+, corn soy blend plus; IFA, iron and folic acid supplement; SQ-LNS, small-quantity lipid-based nutrient supplement; P&L, pregnancy and lactation; ---, not available. ^2^ Reported diet: model energy constraint equal to the reported mean energy intake for each target group; Added meal diet: model energy constraint increased above the report diet to approximate the provision of an “added meal” (~600 kcal) per day among pregnant women or to match estimated energy requirements in lactating women. ^3^ Series modeled with and without lower calcium recommendation (800 mg/day). ^4^ Series which included 1 serving of Supercereal per day also included 1 serving/day of vegetable oil (10.5–10.7 g) and 1–2 servings/day of sugar (6.3–8.5 g) per 100 g Supercereal serving, based on local food patterns and preparation recommendations.

**Table 2:** Demographic characteristics of pregnant and lactating women and their households ^1^.

Variable	Pregnant	Lactating
Participants (*n*)	99	103
Age (years) ^2^	27.8 ± 6.2	26.5 ± 6.4
Gravidity (*n*)	7.2 ± 3.3	---
Current pregnancy trimester		
Second, *n* (%)	59 (59.6)	---
Third, *n* (%)	40 (40.4)	---
Attended ANC in current pregnancy, *n* (%)	65 (65.7)	---
Age of breastfed child (months)	---	8.3 ± 5.6
Menses resumed, *n* (%)	---	26 (25.5)
Household food insecurity access scale (HFIAS), *n* (%)		
Food secure or mildly food insecure	48 (48.5)	43 (42.2)
Moderately food insecure	26 (26.3)	32 (31.3)
Severely food insecure	25 (25.3)	27 (26.5)
Daily per capita reported cost of foods consumed, € ^3^	0.35 (0.28, 0.45)	0.39 (0.30, 0.49)
Daily per capita reported cost of foods below the national poverty line, % ^4^	72.3	63.0
Received food rations in prior month, *n* (%)	6 (10.9) ^5^	3 (2.9)
Adequate minimum dietary diversity – women (MDD-W), *n* (%)	16 (16.3)	15 (14.6)
Nutritional and health status		
Weight (kg)	56.4 ± 8.4	52.4 ± 9.0
BMI (kg/m^2^)	---	20.9 ± 3.2
Underweight (BMI < 18.5 kg/m^2^)	---	22 (21.4)
Overweight (BMI > 25 kg/m^2^)	---	9 (8.7)
Mid-upper arm circumference (cm)	25.1 ± 2.7	26.0 ± 2.9
MUAC < 23 cm	20 (20.3)	3 (2.9)

^1^ ANC, antenatal consultation; HFIAS, household food insecurity access scale; MDD-W, minimum dietary diversity –women; BMI, body mass index; MDD-W, minimum dietary diversity - women; MUAC, mid-upper arm circumference; ---, not available. ^2^ Mean + SD, all such values. **^3^** Median (IQR); values calculated based on reported dietary intakes from 24 h dietary recalls using the National Cancer Institute (NCI) method to adjust for usual intake from [41,42]. ^4^ Cut-off values to define the national poverty line based on daily per capita food consumption expenditures, based on 2400 kcal food baskets and assuming an agro-pastoral system from [49]. ^5^ Data available for only *n* = 55 pregnant women; rations received by pregnant and lactating women included rice, millet, sorghum, beans, small-quantity lipid-based nutrient supplement and Supercereal.

**Table 3:** Median (IQR) usual daily dietary intakes of macro- and micronutrients from foods among pregnant and lactating women and prevalence of inadequate micronutrient intakes ^1^.

	Pregnant Women (*n* = 99)			Lactating Women (*n* = 103)		
	EAR ^2^	Intake	Prevalence of Inadequacy (%)	EAR	Intake	Prevalence of Inadequacy (%)
Energy, kcal	2674.5	1759.7 (1475.5, 2101.3)		2622.2	2209.7 (1841.3, 2640.0)	
Vitamin A, µg RAE	550	536.1 ^3^ (378.4, 741.1)	52.1	900	504.9 (349.8, 701.6) ^3^	88.8
Vitamin C, mg	70	25.9 (16.2, 39.7)	95.2	100	30.8 (19.3, 46.7)	97.9
Thiamin, mg	1.2	0.8 (0.7, 1.0)	89.3	1.2	1.0 (0.8, 1.2)	71.8
Riboflavin, mg	1.2	0.8 (0.6, 1.0)	91.8	1.3	0.9 (0.7, 1.2)	85.7
Niacin, mg	14	7.5 (6.4, 8.9)	98.4	13	9.1 (7.7, 10.8)	95.3
Vitamin B_6_, mg	1.6	1.6 (1.3, 1.9)	52.1	1.7	2.0 (1.7, 2.5)	26.9
Folate, µg DFE	520	307.3 ^3^(221.4, 414.7)	88.8	450	294.1 ^3^ (208.4, 398.8)	83.0
Vitamin B_12_, µg	2.2	0.2 (0.1, 0.4)	100.0	2.4	0.3 (0.2, 0.5)	100.0
Iron, mg	22	22.6 ^3^ (17.5, 28.9)	46.7	11.7	30.1 ^3^ (23.6, 37.4)	1.0
Zinc, mg	9.5	11.0 (8.8, 13.7)	32.6	10.4	14.8 (11.9, 18.3)	14.3
Calcium, mg	800	330.3 (301.5, 361.5)	100.0	800	384.8 (351.0, 420.2)	100.0

^1^ DFE, dietary folate equivalents; EAR, estimated average requirement; kcal, kilocalorie; RAE, retinol activity equivalent. ^2^ Estimated average requirements from [35,36,37,38]. Estimated energy requirement (EER) = 354 − (6.91 × age [year]) + PA × [(9.36 × weight [kg]) + (726 × height [m])] + physiological group adjustment, where physical activity (PA) = 1.27 (active), and physiological group adjustments were as follows: 2nd trimester pregnancy = + 330; 3rd trimester pregnancy = + 452; 0–6 month postpartum = + 330; 7–23 months post-partum = + 400. ^3^ Assumes vitamin A fortification of cooking oil at 11 mg/kg from [31] and folic acid and iron fortification of wheat flour at 2.5 mg/kg and 60 mg/kg, respectively [32]. Does not include iron and folic acid supplements.

**Table 4:** The nutrient content of worst-case and best-case scenario diets without food-based recommendations (module III), and food-based recommendations with the greatest nutritional impact expressed as a percentage of Recommended Dietary Allowances (RDA) among pregnant women ^1,2^.

	% of RDA												
Analysis ^3^	Vitamin A	Vitamin C	Thiamin	Riboflavin	Niacin	Vitamin B_6_	Folate	Vitamin B_12_	Iron	Zinc	Calcium ^4^	No. MN Adequate	Cost of Diet (€/day)
Reported energy intake													
Best-case scenario	79.2	39.6	82.7	78.9	57.6	113.0	70.6	24.7	133.4	177.2	53.5	3	
Worst-case scenario	0	0.1	38.6	23.3	27.9	50.2	7.1	1.8	32.9	78.8	2.4	1	
Reported energy intake + IFA													
Best-case scenario	79.2	39.6	83.4	79.6	57.6	113.5	184.1	25.0	355.9	177.3	53.5	4	
Worst-case scenario	0	0.1	39.3	24.0	27.9	50.7	120.3	2.1	254.8	78.9	2.4	3	
Added meal													
Best-case scenario	111.8	65.6	122.6	121.9	87.0	154.9	116.6	47.1	188.2	267.7	87.4	7	
Worst-case scenario	0	0.1	50.5	25.7	38.1	62.8	9.9	2.4	32.8	100.1	1.4	1	
Best modeled FBR (worst-case scenario)	74.5	26.3	77.5	80.9	52.5	107.4	73.9	39.8	106.7	209.9	66.4	8	0.43
Added meal + IFA													
Best-case scenario	111.8	65.6	123.3	122.7	87.1	155.4	230.1	47.4	410.8	267.8	87.4	7	
Worst-case scenario	0	0.1	51.2	26.4	38.1	63.4	123.1	2.7	254.7	100.2	1.4	3	
Best modeled FBR (worst-case scenario)	74.4	26.1	70.6	79.9	52.2	106.3	167.3	40.0	323.7	205.2	65.1	8	0.42
Added meal + UNIMMAP													
Best-case scenario	215.8	148.0	222.7	222.1	187.2	255.1	230.1	147.2	299.5	404.2	87.4	10	
Worst-case scenario	103.8	82.3	150.3	125.6	137.9	162.7	123.1	102.2	143.8	236.3	1.4	10	
Best modeled FBR (worst-case scenario)	134.1	108.3	169.8	179.0	152.0	205.6	167.3	139.5	212.7	341.3	65.1	11	0.40
Added meal + Supercereal (CSB+)													
Best-case scenario	239.1	151.4	146.3	221.0	132.8	204.6	135.9	123.5	208.3	300.7	136.8	11	
Worst-case scenario	142.4	85.9	73.6	131.1	86.0	117.3	30.1	78.9	66.6	139.9	51.7	9	
Best modeled FBR (worst-case scenario)	152.6	100.5	86.7	140.5	89.9	128.3	71.1	79.2	78.7	145.9	68.9	11	0.19
Added meal + SQ-LNS P & L													
Best-case scenario	215.8	183.4	319.4	319.5	284.9	349.5	229.1	247.2	259.5	532.0	115.0	11	
Worst-case scenario	103.8	117.5	245.5	223.7	235.5	256.8	122.1	201.9	103.9	362.6	29.1	10	
Best modeled FBR (worst-case scenario)	128.0	141.8	248.0	248.5	244.7	278.6	126.9	220.5	112.5	396.6	71.1	11	0.28
Added meal + Plumpy’Mum													
Best-case scenario	226.3	149.2	218.4	221.0	193.1	236.4	243.1	145.7	293.2	384.2	96.7	10	
Worst-case scenario	114.3	83.5	142.3	129.7	144.3	144.5	136.4	100.7	149.5	217.3	11.3	10	
Best modeled FBR (worst-case scenario)	144.5	109.0	146.9	164.3	157.8	166.5	142.2	137.7	158.2	287.3	68.6	11	0.30

^1^ CSB+, corn soy blend plus; IFA, iron and folic acid supplement; MN, micronutrients; SQ-LNS, small-quantity lipid-based nutrient supplement; P&L, pregnancy and lactation. ^2^ Best case scenario: diets sequentially modeled for each micronutrient, which would provide the highest possible amount (expressed as % of the RDA) of that micronutrient. “Problem” nutrients (non-shaded) were defined as nutrients where the nutrient did not achieve 100% of the RDA in the maximized best-case scenario; these are nutrients that will likely remain inadequate in the population given the local food supply and food patterns, even if women were to follow the FBR. Worst-case scenario: diets sequentially modeled for each micronutrient, which would provide the least possible amount (expressed as % of the RDA) of that micronutrient. Dietary adequacy for each nutrient was defined as the worst-case scenario for that nutrient being > 65% of the RDA (shaded); if the worst-case scenario is less than 65% (non-shaded) of the RDA, the nutrient is likely to be inadequate in the population, given local food supply and food patterns. ^3^ Energy constraints, food serving sizes and food consumption patterns are presented in Table 1 and Table 4, and Supplemental Tables S1–S3. Best-modeled FBR are presented in Table 6 for each series. ^4^ Series modeled using calcium RDA of 1000 mg/day.

**Table 5:** The nutrient content of worst-case and best-case scenario diets without food-based recommendations (module III), and food-based recommendations with the greatest nutritional impact, expressed as a percentage of Recommended Dietary Allowances (RDA) among lactating women ^1,2^.

	% of RDA												
Analysis ^3^	Vitamin A	Vitamin C	Thiamin	Riboflavin	Niacin	Vitamin B_6_	Folate	Vitamin B_12_	Iron	Zinc	Calcium ^4^	No. MN Adequate	Cost of Diet (€/day)
Current energy intake													
Best-case scenario	52.3	40.9	95.4	83.3	72.3	128.0	81.7	21.9	245.1	199.8	54.8	3	
Worst-case scenario	0.0	0.0	51.9	29.8	38.0	64.2	11.0	2.1	82.0	96.5	4.0	2	
Additional meal													
Best-case scenario	80.9	67.4	128.0	118.5	97.8	155.3	135.3	40.7	306.4	270.7	89.0	6	
Worst-case scenario	0.0	0.1	53.2	25.4	42.1	61.5	11.7	2.4	59.9	98.1	1.9	1	
Best modeled FBR (worst-case scenario)	13.7	15.1	78.4	69.3	56.7	111.6	69.9	37.0	181.9	207.2	65.4	7	0.43
Additional meal + UNIMMAP													
Best-case scenario	142.6	125.8	228.1	206.1	203.9	250.5	271.4	133.7	473.3	395.9	89.0	10	
Worst-case scenario	61.5	58.3	153.0	112.8	147.8	156.3	147.5	95.1	226.3	222.9	1.9	8	
Best modeled FBR (worst-case scenario)	75.2	73.3	178.2	156.7	162.4	206.5	205.7	129.7	348.3	332.0	65.4	11	0.43
Additional meal + Supercereal (CSB+)													
Best-case scenario	156.4	128.2	151.9	204.7	146.1	201.8	158.3	111.7	325.7	300.3	138.4	11	
Worst-case scenario	84.5	60.8	80.9	117.5	93.8	116.5	37.5	73.4	111.2	139.1	51.9	8	
Best modeled FBR (worst-case scenario)	88.9	69.7	91.3	123.6	97.1	129.1	74.6	73.6	125.2	143.3	69.7	11	0.21
Additional meal + SQ-LNS P & L													
Best-case scenario	142.6	150.9	324.8	291.1	307.4	340.1	270.3	226.6	406.9	513.0	116.6	11	
Worst-case scenario	61.5	83.3	249.5	198.4	251.2	246.3	146.8	187.7	166.5	340.6	29.4	9	
Best modeled FBR (worst-case scenario)	71.5	96.9	251.7	213.3	259.9	265.1	150.4	204.8	174.9	372.5	69.9	11	0.30
Additional meal + Plumpy’Mum													
Best-case scenario	148.8	126.7	224.5	204.9	210.3	232.5	287.0	132.3	454.0	377.5	98.2	10	
Worst-case scenario	67.7	59.1	149.8	116.2	155.3	143.2	165.1	93.7	235.0	209.3	11.5	9	
Best modeled FBR (worst-case scenario)	81.3	73.7	153.0	139.6	168.6	162.4	169.5	128.0	243.4	274.0	67.3	11	0.32

^1^ CSB+, corn soy blend plus; IFA, iron and folic acid supplement; MN, micronutrients; SQ-LNS, small-quantity lipid-based nutrient supplement; P&L, pregnancy and lactation. ^2^ Best case scenario: diets sequentially modeled for each micronutrient, which would provide the highest possible amount (expressed as % of the RDA) of that micronutrient. “Problem” nutrients (non-shaded) were defined as nutrients where the nutrient did not achieve 100% of the RDA in the maximized best-case scenario; these are nutrients that will likely remain inadequate in the population given the local food supply and food patterns, even if women were to follow the FBR. Worst-case scenario: diets sequentially modeled for each micronutrient, which would provide the least possible amount (expressed as % of the RDA) of that micronutrient. Dietary adequacy for each nutrient was defined as the worst-case scenario for that nutrient being > 65% of the RDA (shaded); if the worst-case scenario is less than 65% (non-shaded) of the RDA, the nutrient is likely to be inadequate in the population, given local food supply and food patterns. ^3^ Energy constraints, food serving sizes and food consumption patterns are presented in Table 1 and Table 4, and Supplemental Tables S1–S3. Best-modeled FBR are presented in Table 6 for each series. ^4^ Series modeled using calcium RDA of 1000 mg/day.

**Table 6:** Food-based recommendations for pregnant and lactating women, by model series and intervention product ^1^.

	Pregnant Women	No. MN Adequate ^2^	Lactating Women	No. MN Adequate
Reported diet ^3^	---		---	
Reported diet + IFA (standard of care)	---		---	
Added meal diet	21 servings of DGLV 14 servings of milk 21 servings of cooked beans/lentils/peas 14 servings of millet 21 servings of vitamin A fortified vegetable oil	8	21 servings of DGLV 14 servings of milk 21 servings of cooked beans/lentils/peas 14 servings of millet	7
Added meal diet + IFA	21 servings of DGLV 14 servings of milk 14 servings of cooked beans/lentils/peas 14 servings of millet 21 servings of vitamin A fortified vegetable oil	8	---	
Added meal diet + UNIMMAP	21 servings of DGLV 14 servings of milk 14 servings of cooked beans/lentils/peas 14 servings of millet	11	21 servings of DGLV 14 servings of milk 21 servings of cooked beans/lentils/peas 14 servings of millet	11
Added meal diet + Supercereal (CSB+)	14 servings of DGLV 14 servings of cooked beans/lentils/peas	11	14 servings of DGLV 14 servings of cooked beans/lentils/peas	11
Added meal + SQ-LNS (P&L)	21 servings of DGLV 7 servings of milk	11	21 servings of DGLV 7 servings of milk	11
Added meal diet + Plumpy’Mum	21 servings of DGLV 14 servings of milk	11	21 servings of DGLV 14 servings of milk	11

^1^ CSB+, corn soy blend plus; DGLV, dark green leafy vegetables; IFA, iron and folic acid supplement; MN, micronutrients; SQ-LNS, small-quantity lipid-based nutrient supplement; P&L, pregnancy and lactation; ---, not available. ^2^ Maximum number of micronutrients with the potential to be adequate, *n* = 11. ^3^ Reported diet: model energy constraint equal to the reported mean energy intake for each target group; Added meal diet: model energy constraint increased above the reported diet to approximate the provision of an “added meal” (~600 kcal) per day among pregnant women or to match estimated energy requirements in lactating women, in addition to the best set of food-based recommendations.

## Supplementary Materials

The following are available online at http://www.mdpi.com/2072-6643/11/1/72/s1, Table S1: Nutrient composition of intervention products modeled using the Optifood software tool, Table S2: Recommended Dietary Allowances (RDA) used for analyses, Table S3: Food serving size (g/day) and food consumption patterns (number of servings per week by percentiles) in pregnant and lactating study participants, Table S4: Consumption patterns of food group and food subgroup (number of servings per week by percentiles) in pregnant and lactating study participants, Table S5: Market availability and prices of foods from 55 markets surveys conducted at 10 markets in the study area over a period of 18 months, Table S6: The nutrient content of optimal diets with and without considering reported food patterns (module II), expressed as a percentage of Recommended Dietary Allowances (RDA) among pregnant women, Table S7: The nutrient content of optimal diets with and without food patterns (module II), expressed as a percentage of Recommended Dietary Allowances (RDA) among lactating women, Table S8: The nutrient content of worst-case and best-case scenario diets without food-based recommendations (module III), and food-based recommendations with the greatest nutritional impact expressed as a percentage of Recommended Dietary Allowances (RDA) among pregnant and lactating women, Table S9: The nutrient content of worst-case and best-case scenario diets without food-based recommendations (module III), and food-based recommendations with the greatest nutritional impact expressed as a percentage of Recommended Dietary Allowances (RDA) among pregnant women, using lower calcium recommendations (775 mg/day), Table S10: The nutrient content of worst-case and best-case scenario diets without food-based recommendations (module III), and food-based recommendations with the greatest nutritional impact expressed as a percentage of Recommended Dietary Allowances (RDA) among lactating women, using lower calcium recommendations (800 mg/day), Table S11: Food-based recommendations for pregnant and lactating women using lower calcium recommendations (775–800 mg/day), by model series and intervention product.

## Abbreviations

AMDR	acceptable macronutrient distribution range
CSB+	corn soy blend plus
DGLV	dark green leafy vegetables
DRI	dietary reference intake
EAR	estimated average requirement
ECVMA	National Survey on Living Conditions, Household and Agriculture
EER	estimated energy requirement
FBR	food based recommendation
FNB	Food and Nutrition Board
HFIAS	household food insecurity access scale
IFA	iron and folic acid
IOM	Institute of Medicine
MDD-W	minimum dietary diversity for women
MN	micronutrients
MUAC	mid-upper arm circumference
NCI	National Cancer Institute
NiMaNu	Niger Maternal Nutrition Project
PAL	physical activity level
RDA	recommended dietary allowance
SQ-LNS P&L	small quantity lipid-based nutrient supplement for pregnant and lactating women
UEMOA	West African Economic and Monetary Union
UNIMMAP	UNICEF/WHO/UNU international multiple micronutrient preparation
USDA	United States Department of Agriculture
USDA SR28	USDA Nutrient Database for Standard Reference, Release 28
WHO	World Health Organization
