# Supplementary material for: Assessment of Dietary Intake and Nutrient Gaps, and Development of Food-Based Recommendations, among Pregnant and Lactating Women in Zinder, Niger: An Optifood Linear Programming Analysis
Source: Nutrients. 2019 Jan 2;11(1):72. doi: 10.3390/nu11010072 (PMC6357040; doi:10.3390/nu11010072)
Supplement: Supplementary file 1 [file nutrients-11-00072-s001.pdf]

Table S1. Nutrient composition of intervention products modeled using the Optifood software tool[1].

Nutrient	Iron and folic acid (IFA)	UNIMMAP	SQ-LNS P&L	Supercereal (CSB +)[2] Premix + Intrinsic	Plumpy'Mum
Dose, g			20	100	92
Energy, kcal			118	380	515
Carbohydrate, g				64.3	33.1
Fat, g			10	2.5	36.6
Protein, g			2.6	13.3	14.4
Vitamin A, µg RAE		800	800	980	881
Vitamin C, mg		70	100	91	71
Thiamin, mg		1.4	2.8	0.7	1.6
Riboflavin, mg		1.4	2.8	1.7	1.6
Niacin, mg		18	36	11.2	21
Vitamin B-6, mg		1.9	3.8	1.6	2
Folate, µg DFE[3]	680	680	680	200	783
Vitamin B-12, µg		2.6	5.2	2.0	2.6
Iron, mg	60	30	20	11.8	35
Zinc, mg		15	30	7.7	17
Calcium, mg		0	280	512	111

[1] CSB+, corn soy blend plus; DFE, dietary folate equivalents; IFA, iron and folic acid supplement; SQ-LNS, small-quantity lipid-based nutrient supplement; P&L, pregnancy and lactation; RAE, retinol activity equivalent. [2] Nutrient composition of premix and intrinsic ingredients from [https://www.usaid.gov/what-we-do/agriculture-and-food-security/food-assistance/resources/implementation-tools/corn-soy]; USDA retention factors for cooking were applied to appropriate micronutrients [29]. Models which included 1 serving of Supercereal per day also included 1 serving/day of vitamin A fortified vegetable oil (10.5 – 10.7 g) and 1-2 servings/day of sugar (6.3 – 8.5 g) per 100g Supercereal serving, based on local food patterns. [3] IFA, UNIMMAP and SQ-LNS P&L contain 400 µg folic acid, Plumpy'Mum contains 461 µg folic acid; it was assumed that all intervention products were taken with food or fortified food (DFE = µg folic acid * 1.7) [38].

Table S2. Recommended Dietary Allowances (RDA) used for analyses[1,2].

Nutrient	Pregnant women	Lactating women
Energy, kcal/d	2674.5	2622.2
Carbohydrate, g/d	---	---
Fat, % of total energy	20%	20%
Protein, g/d	61.8	68.4
Vitamin A, μg RAE/d	770	1300
Vitamin C, mg/d	85	120
Thiamin, mg/d	1.4	1.4
Riboflavin, mg/d	1.4	1.6
Niacin, mg/d	18	17
Vitamin B-6, mg/d	1.9	2.0
Folate, μg DFE/d	600	500
Vitamin B-12, μg/d	2.6	2.8
Iron, mg/d	27	18
Zinc, mg/d	11	12
Calcium, mg/d	1000	1000

[1] DFE, dietary folate equivalents; RAE, retinol activity equivalent; RDA, recommended dietary allowance. [2]Data from [34-38], unless otherwise noted. Estimated energy requirement (EER) = 354 – (6.91 x age [y]) + PA x {(9.36 x weight [kg]) + (726 x height [m])} + physiological group adjustment, where physical activity (PA) = 1.27 (active), and physiological group adjustments were as follows: 2nd trimester pregnancy = + 330; 3rd trimester pregnancy = + 452; 0-6 month postpartum = +330; 7-23 months post-partum = + 400. Fat recommendation was set at the lower end of the acceptable range (20-35%) to increase likelihood of requirement being met. Protein recommendation: pregnant women = 1.1 * body weight (kg); lactating women = 1.3 * body weight (kg). Iron recommendation assumed 25% absorption during 2nd and 3rd trimesters for pregnant women, 10% absorption for lactating women [3, 39]. Models were also run using calcium recommendations (800 mg/d) from the WHO/FAO for settings where animal source foods provide less than 20-40 g/d of protein [36].

Table S3. Food serving size (g/day) and food consumption patterns (number of servings per week by percentiles) in pregnant and lactating study participants[1].

	Pregnant women (n=113 24h recalls)				Lactating women (n=122 24h recalls)			
			Frequency (servings/week)				Frequency (servings/week)	
	n (%) of recalls consumed	Median serving size (g/serving)	Maximum (90th percentile)[2]	Maximum (adjusted for added meal)[3]	n (%) of recalls consumed	Median serving size (g/serving)	Maximum (90th percentile)[2]	Maximum (adjusted for added meal)[3]
Added fats								
Palm oil	106 (93.8)	10.5	21	24	114 (93.4)	10.7	21	28
Peanut oil	11 (9.7)	7.7	1	2	7 (5.7)	5.6	1	2
Added sugars								
Sugar, granulated	13 (11.5)	3.2	1	2	21 (17.2)	8.5	3	6
Dairy products								
Milk, curdled, whole	23 (20.4)	106.9	7	14	36 (29.5)	106.9	7	14
Grains and grain products[4]								
Pearl millet flour	83 (73.4)	183.2	14	21	104 (85.2)	236.1	15	21
Sorghum flour	16 (14.2)	168.4	6	12	31 (25.4)	190.2	11	17
Maize flour	26 (23.0)	204.0	7	14	28 (23.0)	209.0	7	14
Rice, white, boiled	68 (60.2)	277.8	7	14	56 (45.9)	258.8	5	10
Wheat flour, white	18 (15.9)	65.7	2	4	20 (16.4)	43.8	3	6
Macaroni, wheat	11 (9.7)	100.0	1	2	13 (10.7)	134.0	1	2
Legumes, nuts and seeds								
Cowpeas, dried, boiled	84 (74.3)	57.3	13	21	84 (68.9)	43.2	12	21
Cowpeas, fresh, boiled	6 (5.3)	119.2	1	2	7 (5.7)	245.0	1	2
Groundnuts	7 (6.2)	12.7	1	2	8 (6.6)	5.9	1	2
Groundnut flour, defatted	6 (5.3)	6.8	1	2	---	---	---	---
Locust bean, fermented	78 (69.0)	2.5	14	21	94 (77.0)	2.4	14	21
Soy flour	13 (11.5)	15.9	2	4	9 (7.4)	29.3	1	2
Meat, fish & eggs								
Lamb, cooked	7 (6.2)	13.1	1	2	---	---	---	---
Vegetables								

Baobab leaves, dried	46 (40.7)	5.3	10	15	54 (44.3)	3.9	14	21
False sesame, dried	7 (6.2)	7.4	1	2	---	---	---	---
Jute leaves, fresh	29 (25.7)	29.1	4	8	41 (33.6)	34.2	7	14
Roselle leaves, fresh	20 (17.7)	33.4	2	4	19 (15.6)	22.2	2	4
Leaves, other, boiled	7 (6.2)	66.7	1	2	---	---	---	---
Garlic, fresh	47 (41.6)	0.5	10	15	48 (39.3)	0.5	14	21
Onion, fresh	79 (69.9)	6.8	14	21	75 (61.5)	7.2	18	24
Okra, dried	10 (8.8)	2.3	1	2	10 (8.2)	6.3	1	2
Peppers, chili, fresh	45 (39.8)	1.4	7	14	47 (38.5)	2.5	14	21
Tomato, fresh	15 (13.3)	6.4	1	2	14 (11.5)	7.2	2	4
Tomato, dried	50 (44.2)	2.5	14	21	63 (51.6)	2.6	18	24
Tomato, paste	39 (34.5)	3.4	7	14	38 (31.1)	3.9	14	21
Squash, orange flesh, fresh	12 (10.6)	23.4	1	2	24 (19.7)	33.2	3	6
Miscellaneous								
Pepper, black	37 (32.7)	0.3	7	14	43 (35.2)	0.4	14	21
Salt, table	108 (95.6)	2.6	21	28	114 (93.4)	2.9	21	28
Peppers, chili, dried	23 (20.4)	2.4	2	4	26 (21.3)	0.8	5	10
Bouillon cubed, dry	99 (87.6)	1.4	16	21	116 (95.1)	1.4	21	28

[1]Foods consumed by >5% of participants are considered "common foods" and included in this list. All values are presented based on edible portion only, and assume the food is raw, unless otherwise noted. [2] Maximum frequency was defined as the 90th percentile of the food frequency distribution for each group (pregnant and lactating women), with a lower limit of 1 serving/week and an upper limit of 21 servings/week. [3] Maximum frequency (adjusted for an additional meal) depended on the reported consumption frequency at the 90th percentile, such that the maximum allowable frequency of consumption was proportionate to the reported consumption, with an upper limit at the 100th percentile of reported consumption. [4] Median serving sizes (g/d) for staple foods (grains and grain products) were defined as the 75th percentile, to allow for adequate flexibility to modeled energy.

Table S4. Consumption patterns of food groups and food subgroups (number of servings per week by percentiles) in pregnant and lactating study participants.

	Pregnant women			Lactating women		
	Frequency (servings/week)			Frequency (servings/week)		
	Median (50th percentile)	Maximum (90th percentile)[1]	Maximum (additional meal)	Median (50th percentile)	Maximum (90th percentile)	Maximum (additional meal)
Added fats	13	21	24	12.5	21	28
Vegetable oil		21	24		21	28
Added sugars	0.1	1	2	0.1	3	6
Sugar		1	2		3	6
Dairy products	0.1	7	14	0.1	7	14
Fluid or powdered milk		7	14		7	14
Grains and grain products	11	19	28	14	23	28
Refined grains and products		18	24		21	28
Whole grains and products		7	14		7	14
Legumes, nuts and seeds	10	22	28	10	27	36
Cooked beans, lentils, peas		13	21		12	18
Nuts, seeds and unsweetened products		14	21		16	21
Soybeans and products		2	4		1	2
Meat, fish & eggs	0.1	1	2	---	---	---
Red meat		1	2		---	---
Vegetables	15	57	75	21.5	69	92
Vitamin A source dark green leafy vegetables		14	21		14	21
Vitamin A source other vegetables		1	2		3	6
Condiment vegetables		42	56		56	75
Other vegetables		2	4		3	6
Miscellaneous	25	42	56	27	56	75
Condiments, herbs, spices		42	56		56	75

[1] Maximum frequency was defined as the 90th percentile of the food frequency distribution for each group (pregnant and lactating women), with a lower limit of 1 serving/week and no upper limit to servings/week. [2] Maximum frequency (adjusted for an additional meal) depended on the reported consumption frequency at the 90th percentile, such that the maximum allowable frequency of consumption was proportionate to the reported consumption, with an upper limit at the 100th percentile of reported consumption.

Table S5. Market availability and prices of foods from 55 market surveys conducted at 10 markets in the study area over a period of 18 months[1].

	Availability, n (%)	Median price (CFA/100g)

	June – Sept	Oct – Feb	Mar – May	Overall	June – Sept	Oct – Feb	Mar – May	Overall
Added fats								
Butter	9 (29.0)	1 (9.1)	1 (8.3)	11 (20.4)	177.8 (120.0, 181.8)	344.4	177.8	177.8 (120.0, 244.4)
Margarine, fortified	9 (29.0)	1 (9.1)	1 (8.3)	11 (20.4)	181.8 (150.0, 200.0)	420.0	120.0	181.8 (120.0, 320.0)
Palm oil, fortified[2]	25 (80.7)	10 (90.9)	10 (83.3)	45 (83.3)	81.3 (81.3, 89.3)	81.3 (81.3, 92.1)	82.7 (81.3, 86.7)	81.3 (81.3, 86.7)
Peanut oil, fortified	27 (87.1)	9 (81.8)	11 (91.7)	47 (87.0)	130.1 (108.4, 137.9)	108.4 (97.6, 108.4)	119.2 (104.2, 130.1)	119.2 (100.0, 130.1)
Dairy products								
Milk, curdled, whole	27 (87.1)	6 (54.6)	8 (66.7)	41 (75.9)	50.0 (33.3, 86.4)	62.2 (31.6, 89.3)	41.2 (28.0, 48.0)	49.0 (31.6, 74.6)
Milk, powdered	31 (100)	9 (81.8)	12 (100)	52 (96.3)	380.0 (340.9, 400.0)	350.0 (350.0, 420.0)	365.0 (345.0, 390.0)	380.0 (347.5, 400.0)
Grains and grain products								
Maize	30 (93.8)	10 (90.9)	12 (100)	52 (94.6)	17.8 (17.0, 20.3)	21.1 (15.8, 24.6)	17.8 (16.7, 19.7)	18.0 (16.8, 20.7)
Pearl millet (*Pennisetum glaucum*)	32 (100)	10 (90.9)	12 (100)	54 (98.2)	27.1 (25.1, 32.1)	29.9 (25.5, 34.4)	28.1 (22.8, 30.4)	27.5 (25.1, 31.9)
Pearl millet (*Pennisetum americanum*)	16 (50.0)	3 (27.3)	5 (41.7)	24 (43.6)	30.1 (23.4, 86.9)	12.2 (11.6, 29.5)	26.1 (23.8, 27.9)	27.0 (22.6, 72.9)
Sorghum, red	29 (90.6)	8 (72.7)	11 (91.7)	48 (87.3)	25.7 (21.9, 29.0)	21.6 (16.5, 27.6)	24.2 (20.6, 25.6)	25.1 (20.9, 27.8)
Sorghum, white	25 (78.1)	8 (72.3)	10 (83.3)	43 (78.2)	26.4 (21.7, 29.8)	23.8 (15.7, 25.2)	23.8 (20.0, 26.2)	25.1 (21.4, 28.6)
Rice, white	31 (96.9)	10 (90.9)	11 (91.7)	52 (94.6)	50.0 (50.0, 50.0)	50.0 (50.0, 55.0)	50.0 (50.0, 54.8)	50.0 (50.0, 50.0)
Wheat, couscous	28 (87.5)	7 (63.6)	9 (75.0)	44 (80.0)	61.25 (60.0, 65.0)	65.0 (62.5, 75.0)	65.0 (64.4, 85.0)	65.0 (60.0, 67.5)
Wheat, flour, white	27 (84.4)	8 (72.7)	8 (66.7)	43 (78.2)	40.0 (40.0, 44.6)	43.9 (40.0, 50.5)	40.0 (40.0, 45.1)	40.0 (40.0, 45.0)
Wheat, macaroni	32 (100)	11 (100)	12 (100)	55 (100)	65.0 (60.0, 65.0)	65.0 (65.0, 65.0)	65.0 (61.2, 72.5)	65.0 (60.0, 65.0)
Legumes, nuts and seeds								

Cowpeas, dried	31 (96.9)	11 (100)	11 (91.7)	53 (96.4)	40.3 (34.7, 53.0)	30.9 (27.2, 38.2)	40.0 (31.5, 51.0)	37.3 (32.4, 50.0)
Groundnuts[3]	28 (87.5)	6 (54.6)	9 (75.0)	43 (78.2)	74.0 (67.6, 89.3)	67.6 (63.7, 108.7)	73.6 (55.9, 109.0)	71.7 (63.7, 108.7)
Groundnuts, butter	21 (65.6)	8 (72.7)	9 (75.0)	38 (69.1)	92.6 (47.2, 111.1)	68.2 (37.0, 112.1)	83.3 (80.6, 108.7)	83.3 (47.2, 111.1)
Sesame seed	11 (34.4)	5 (45.5)	2 (16.7)	18 (32.7)	64.5 (58.1, 113.6)	108.7 (57.8, 111.1)	54.8 (42.9, 66.7)	65.6 (57.8, 111.1)
Locust bean, fermented	31 (96.9)[a]	6 (54.6)[b]	9 (75.0)[a,b]	46 (83.6)	178.6 (100.0, 263.2)	179.5 (156.3, 222.2)	166.7 (138.9, 250.0)	172.6 (111.1, 250.0)
Soybeans, dried	18 (56.3)	4 (36.4)	5 (41.7)	27 (49.1)	56.0 (37.8, 60.0)	49.1 (46.3, 71.7)	47.1 (40.0, 56.4)	50.8 (38.8, 60.0)
Starchy Roots								
Cassava	30 (100)	11 (100)	11 (91.7)	52 (98.1)	40.4 (37.2, 50.4)	36.7 (24.2, 50.4)	39.2 (32.3, 58.6)	39.7 (32.3, 50.4)
Potato, white flesh	11 (34.4)	2 (18.2)	5 (41.7)	18 (32.7)	71.4 (41.7, 107.1)	59.5 (59.5, 59.5)	56.2 (47.6, 59.2)	59.5 (47.6, 93.6)
Sweet potato, white flesh	17 (53.1)	8 (72.7)	9 (75.0)	34 (61.8)	28.6 (23.8, 49.6)	27.7 (16.8, 41.1)	30.4 (20.1, 45.6)	29.2 (18.5, 45.6)
Yam	9 (28.1)	7 (63.6)	2 (16.7)	18 (32.7)	112.2[a] (40.3, 125.3)	31.8[b] (30.2, 40.5)	49.5[a,b] (43.2, 55.8)	41.8 (36.2, 112.2)
Meat, fish & eggs								
Beef, fresh, raw	11 (34.4)	3 (27.3)	4 (33.3)	18 (32.7)	138.2 (109.9, 166.7)	222.2 (192.7, 274.0)	145.8 (119.3, 176.8)	146.8 (125.6, 167.8)
Beef liver, fresh, raw	10 (31.3)	2 (18.2)	2 (16.7)	14 (25.5)	223.1 (211.9, 360.0)	230.4 (161.5, 299.4)	342.0 (310.6, 373.4)	266.0 (211.9, 360.0)
Beef, offal, raw	9 (28.1)	3 (27.3)	3 (25.0)	15 (27.3)	226.9 (200.0, 238.6)	221.2 (109.7, 253.4)	227.3 (178.6, 247.5)	226.9 (178.6, 242.7)
Chicken, whole[4]	28 (87.5)	10 (90.9)	9 (75.0)	47 (85.5)	239.0 (191.2, 274.9)	239.0 (191.2, 286.8)	191.2 (167.3, 239.0)	239.0 (191.2 , 286.8)
Crickets, fresh, raw	9 (28.1)	3 (27.3)	4 (33.3)	16 (29.1)	316.1 (186.5, 459.2)	169.8 (90.1, 217.4)	220.0 (130.5, 419.6)	214.0 (163.0, 387.6)
Duck, whole[4]	19 (59.4)	8 (72.7)	9 (75.0)	36 (65.5)	236.6 (197.2, 276.0)	197.2 (118.3, 285.9)	197.2 (157.7, 276.0)	216.9 (157.7, 276.0)
Goat, fresh, raw	28 (87.5)	10 (90.9)	12 (100)	50 (90.9)	140.6	216.6	150.6	150.6

					(116.3, 161.7)	(134.0, 378.8)	(99.4, 187.0)	(116.3, 187.3)
Goat liver, fresh, raw	26 (81.3)	7 (63.6)	8 (66.7)	41 (74.6)	268.1 (196.9, 369.5)	324.7 (187.5, 437.2)	227.8 (150.5, 373.4)	261.5 (196.1, 370.4)
Goat offal, raw	26 (81.3)	8 (72.7)	10 (83.3)	44 (80.0)	151.7 (103.0, 240.0)	178.8 (141.5, 357.4)	144.4 (125.0, 229.4)	157.7 (122.1, 239.6)
Guinea fowl, whole[4]	28 (87.5)	10 (90.9)	9 (75.0)	47 (85.5)	322.7 (286.8, 382.4)	286.8 (286.8, 334.6)	274.9 (227.1, 298.8)	286.8 (286.8, 334.6)
Pigeon, whole[4]	20 (62.5)	9 (81.8)	5 (41.7)	34 (61.8)	251.3 (226.1, 301.5)	150.8 (125.6, 251.3)	251.3 (150.8, 251.3)	251.3 (175.9, 301.5)
Mutton, fresh	19 (59.4)	5 (45.5)	5 (41.7)	29 (52.7)	152.9 (133.3, 187.5)	155.8 (137.0, 300.0)	202.0 (151.5, 300.0)	155.0 (135.2, 216.4)
Mutton, liver, fresh, raw	17 (53.1)	5 (45.5)	2 (16.7)	24 (43.6)	300.3 (200.0, 365.6)	169.1 (112.4, 404.4)	326.5 (266.7, 386.4)	283.5 (193.1, 378.4)
Mutton, offal, raw	19 (59.4)	5 (45.5)	3 (25.0)	27 (49.1)	142.0 (105.9, 185.2)	207.8 (121.5, 314.7)	144.8 (106.7, 566.0)	144.8 (106.7, 207.8)
Fish, fresh, smoked or dried[5]	16 (50.0)	5 (45.5)	7 (58.3)	28 (50.9)	---	---	---	---
Fish, canned tuna	25 (80.7)	9 (81.8)	11 (91.7)	45 (83.3)	260.0 (250.0, 280.0)	280.0 (260.0, 280.0)	260.0 (209.7, 280.0)	260.0 (240.0, 280.0)
Egg, chicken, raw	29 (93.6)[a]	2 (18.2)[b]	4 (33.3)[b]	35 (64.8)	179.0 (149.5, 224.3)	354.2 (214.4, 494.1)	206.1 (112.9, 311.9)	181.8 (151.5, 233.5)
Vegetables[6]								
Vitamin A source dark green leafy vegetables								
Baobab leaves, dried	30 (93.8)	10 (90.9)	10 (83.3)	50 (90.9)	46.3 (35.1, 69.4)	58.2 (31.6, 73.5)	40.9 (35.2, 54.0)	46.3 (35.1, 69.4)
Jute leaves, dried	26 (81.3)	7 (63.6)	6 (50.0)	39 (70.9)	63.6 (29.4, 75.8)	86.2 (12.9, 121.0)	42.9 (26.0, 75.8)	63.6 (29.3, 80.7)
Moringa leaves, fresh, boiled	9 (28.1)	0 (0.0)	3 (25.0)	12 (21.8)	46.7 (37.3, 54.1)	---	54.5 (34.7, 62.5)	48.9 (36.0, 58.5)
Moringa leaves, dried	11 (34.4)	4 (36.4)	1 (8.3)	16 (29.1)	125.0 (93.8, 190.6)	49.3 (25.5, 83.2)	114.9	112.4 (70.3, 172.5)
Roselle leaves, fresh	25 (78.1)[a]	4 (36.4)[b]	9 (75.0)[a]	38 (69.1)	21.6 (16.0, 28.4)	30.3 (23.7, 45.9)	22.3 (13.1, 31.3)	22.3 (16.0, 32.6)
Roselle leaves, dried	21 (65.6)	6 (54.6)	10 (83.3)	37 (67.3)	51.5 (30.1, 78.1)	41.4 (31.7, 53.2)	41.0 (34.7, 67.6)	49.5 (30.1, 73.5)

Leaves, other, fresh, boiled	13 (40.6)	0 (0.0)	3 (25.0)	16 (29.1)	50.0 (24.6, 100.0)	---	106.9 (33.7, 180.2)	50.0 (24.6, 100.0)
Lettuce, fresh	13 (40.6)	6 (54.6)	9 (75.0)	28 (50.9)	79.0 (31.1, 110.2)	41.1 (20.6, 95.9)	29.6 (16.6, 40.8)	44.1 (27.7, 94.9)
Vitamin A source other vegetables								
Carrots, fresh	7 (21.9)	3 (27.3)	7 (58.3)	17 (30.9)	102.1 (99.1, 107.0)	43.2 (19.2, 80.3)	27.0 (15.7, 89.2)	80.3 (27.0, 102.1)
Squash, orange flesh, fresh	27 (84.4)	8 (72.7)	12 (100)	47 (85.5)	33.2[a] (24.1, 38.2)	21.6[a,b] (16.8, 48.1)	15.2[b] (9.6, 26.1)	26.5* (18.8, 35.0)
Vitamin C rich vegetables								
Okra, fresh	11 (34.4)	0 (0.0)	2 (16.7)	13 (23.6)	110.7 (83.1, 122.8)	---	135.0 (84.9, 185.2)	110.7 (84.9, 122.8)
Okra, dried	26 (81.3)	10 (90.9)	11 (91.7)	47 (85.5)	185.2 (65.8, 333.3)	129.0 (57.0, 217.4)	185.2 (122.0, 303.0)	179.5 (80.6, 303.0)
Cabbage, fresh	11 (34.4)	5 (45.5)	8 (66.7)	24 (43.6)	56.8[a] (27.8, 106.3)	33.2[a,b] (30.1, 45.1)	13.2[b] (9.6, 27.1)	29.0* (20.0, 58.1)
Peppers, sweet, fresh	10 (31.3)	2 (18.2)	4 (33.3)	16 (29.1)	148.8 (143.4, 200.8)	152.9 (54.8, 251.0)	139.9 (88.0, 198.1)	148.8 (96.6, 209.9)
Zucchini, green, fresh	15 (46.9)	2 (18.2)	5 (41.7)	22 (40.0)	15.5 (14.6, 28.3)	13.7 (9.9, 17.5)	13.6 (11.5, 15.3)	15.5 (13.4, 23.1)
Other vegetables								
Eggplant, fresh	10 (31.3)	2 (18.2)	4 (33.3)	16 (29.1)	54.0 (42.1, 104.6)	55.0 (10.4, 99.6)	44.6 (41.3, 47.1)	47.4 (40.6, 93.9)
Tomato, fresh	12 (37.5)	7 (63.6)	12 (100)	31 (56.4)	70.4[a] (58.5, 123.5)	35.2[b] (28.9, 44.6)	31.5[b] (14.3, 55.2)	53.2* (29.8, 69.4)
Condiment vegetables								
Onion, fresh	31 (96.9)	11 (100)	12 (100)	54 (98.2)	27.9[a,b] (20.2, 49.1)	53.5[a] (36.6, 61.1)	20.9[b] (13.1, 32.2)	31.9* (20.2, 53.5)
Tomato, dried, powdered	29 (90.6)[a]	3 (27.3)[b]	10 (83.3)[a]	42 (76.4)	125.0 (70.1, 162.0)	35.3 (34.9, 238.1)	123.8 (92.6, 161.3)	125.0 (71.8, 161.3)
Tomato, paste	32 (100)	10 (90.9)	11 (91.7)	53 (96.4)	142.9 (125.0, 145.0)	142.9 (133.3, 151.5)	142.9 (108.7, 178.6)	142.9 (125.0, 151.5)
Fruits[6]								
Vitamin C rich fruit								
Baobab fruit, fresh, raw	13 (40.3)	5 (45.5)	2 (16.7)	20 (36.4)	188.6 (144.0, 443.3)	129.4 (128.5, 209.5)	268.5 (148.8, 388.2)	174.0 (137.4, 356.0)

Citrus, fresh, raw (lemon, lime, grapefruit)	24 (75.0)	6 (54.6)	5 (41.7)	35 (63.6)	100.2 (86.8, 126.0)	85.6 (62.0, 111.6)	88.8 (83.1, 156.3)	98.9 (80.2, 126.0)
Guava, fresh, raw	10 (31.3)	1 (9.1)	0 (0)	11 (20.0)	58.7 (44.2, 118.7)	32.7	---	50.6 (33.7, 118.7)
Jujube, fresh, raw	9 (28.1)	1 (9.1)	6 (50.0)	16 (29.1)	23.9 (11.3, 28.9)	23.8	18.0 (17.9, 21.5)	21.6 (14.6, 26.1)
Mango, fresh, raw	10 (32.3)	2 (18.2)	12 (100)	24 (44.4)*	41.8 (32.5, 43.9)	51.3 (15.6, 86.9)	29.5 (21.4, 40.0)	35.6 (23.1, 43.9)
Orange, fresh, raw	21 (67.7)	5 (45.5)	9 (75.0)	35 (64.8)	66.7 (48.7, 69.9)	48.0 (42.3, 82.9)	37.8 (31.1, 48.7)	54.1 (37.8, 69.9)
Palm fruit, fresh, raw	16 (50.0)	3 (27.3)	4 (33.3)	23 (41.8)	41.4 (19.1, 103.3)	51.1 (25.1, 54.3)	28.9 (16.5, 44.7)	40.9 (19.0, 54.3)
Pineapple, fresh raw	11 (35.5)	2 (18.2)	2 (16.7)	15 (27.8)	146.1 (101.6, 198.1)	139.7 (111.4, 168.1)	202.5 (90.7, 314.2)	146.1 (101.6, 198.1)
Other fruit								
Banana, ripe, fresh, raw	12 (38.7)	3 (27.7)	2 (16.7)	17 (31.5)	116.3 (78.3, 128.9)	116.6 (97.0, 153.2)	59.3 (53.3, 65.1)	115.3 (78.2, 124.8)
Dates, fresh, raw	29 (90.6)	8 (72.7)	10 (83.3)	45 (85.5)	73.1[a,b] (61.7, 111.1)	104.4[a] (92.9, 126.1)	52.8[b] (45.2, 63.1)	77.0 (60.4, 106.8)
Desert date, fresh, raw	18 (56.3)	4 (36.4)	3 (25.0)	25 (45.5)	18.5[a,b] (16.0, 24.0)	24.6[a] (20.2, 33.4)	12.5[b] (5.8, 16.0)	18.4 (16.0, 24.0)
Tamarind, fresh, raw	30 (93.8)	10 (90.9)	11 (91.7)	51 (92.7)	76.0 (66.5, 95.0)	80.6 (54.3, 84.4)	73.9 (38.0, 84.4)	75.4 (56.6, 84.4)
Watermelon, fresh, raw	18 (56.3)	3 (27.3)	6 (50.0)	27 (49.1)	73.6[a] (68.7, 169.2)	36.4[a,b] (32.5, 45.3)	30.4[b] (23.8, 69.2)	69.5 (35.6, 106.3)
Miscellaneous								
Salt, table	32 (100)	11 (100)	12 (100)	55 (100)	24.3 (18.8, 26.9)	24.8 (16.6, 27.8)	23.1 (20.1, 25.8)	24.3 (18.8, 26.3)
Bouillon cubed, dry	32 (100)	11 (100)	12 (100)	55 (100)	250.0 (200.0, 250.0)	250.0 (250.0, 300.0)	250.0 (229.2, 300.0)	250.0 (204.2, 300.0)

[1]Data are presented for foods recorded as being available in at least 11 of the 55 market surveys completed. [2] Includes data for vegetable oil, not otherwise specified. [3] Availability data are for groundnuts (shelled or unshelled); price data are based on the less expensive of the two forms (edible portion only), if both forms were recorded as being available in the same market survey.[4] Poultry weights are from [27], edible portion only (chicken and guinea fowl: 1,046 g; duck: 1,268 g; pigeon: 199 g). [5] Availability data are for fish (fresh, smoked or dried); price data were variable depending on source and preparation and of insufficient sample size to present for one type only. [6] Food groups and sub-groups are defined in the Optifood software tool; vitamin A source vegetables have ≥ 60 RAE/100 g edible portion, vitamin C-rich fruits and vegetables have ≥ 18 mg/100 grams edible portion.

Table S6. The nutrient content of optimal diets with and without considering reported food patterns (module II), expressed as a percentage of Recommended Dietary Allowances (RDA) among pregnant women[1,2].

**Analysis**[3]	**% of RDA**										
	Vitamin A	Vitamin C	Thiamin	Riboflavin	Niacin	Vitamin B_6	Folate	Vitamin B_{12}	Iron	Zinc	Calcium[4]
**Reported energy intake**											
Optimal diet, with food patterns	49.8	24.7	65.0	56.8	40.8	97.9	40.8	3.8	100.0	125.5	29.1
Optimal diet, without food patterns	65.0	38.4	71.6	69.7	48.7	86.2	67.9	23.8	100.0	148.9	47.9
**Reported energy intake + IFA**											
Optimal diet, with food patterns	48.1	25.7	68.0	53.1	42.9	100.0	153.9	4.2	308.2	122.4	29.4
Optimal diet, without food patterns	76.9	39.2	66.6	70.3	44.8	96.3	167.5	24.1	328.6	157.8	50.9
**Added meal**											
Optimal diet, with food patterns	61.5	32.2	93.7	72.9	62.1	121.6	47.4	4.9	108.0	164.0	26.5
Optimal diet, without food patterns	100.0	64.9	100.0	100.0	72.0	110.7	100.0	45.4	105.3	215.6	80.7
**Added meal + IFA**											
Optimal diet, with food patterns	60.7	33.6	94.7	73.7	61.8	121.9	160.0	4.8	323.2	163.9	25.9
Optimal diet, without food patterns	100.0	64.8	100.0	100.0	73.4	113.8	199.5	45.7	323.2	216.4	80.1
**Added meal + UNIMMAP**											
Optimal diet, with food patterns	146.7	101.4	188.8	158.5	161.5	236.4	154.3	104.0	203.2	288.0	37.8
Optimal diet, without food patterns	188.7	130.0	194.1	202.5	165.9	230.0	219.0	145.3	261.9	368.8	86.6
**Added meal + Supercereal (CSB+)**											

Optimal diet, with food patterns	174.0	83.6	119.4	157.5	109.9	187.3	72.3	80.8	123.5	194.8	83.6
Optimal diet, without food patterns	188.2	110.3	127.2	160.0	110.7	187.0	99.0	99.0	103.6	223.7	99.0
**Added meal + SQ-LNS P & L**											
Optimal diet, with food patterns	147.0	137.0	285.8	255.9	259.7	331.0	154.0	204.3	159.2	416.1	65.5
Optimal diet, without food patterns	160.9	156.8	304.8	278.8	269.3	331.2	201.4	231.8	153.2	480.0	99.0
**Added meal + Plumpy'Mum**											
Optimal diet, with food patterns	157.6	102.8	185.9	157.4	168.9	217.7	169.3	102.8	190.6	268.3	47.1
Optimal diet, without food patterns	164.3	131.3	197.2	206.6	177.1	221.8	234.9	144.1	262.3	365.1	96.7

[1]CSB+, corn soy blend plus; IFA, iron and folic acid supplement; SQ-LNS, small-quantity lipid-based nutrient supplement; P&L, pregnancy and lactation. [2] **Optimal diet, with food patterns**: Diet that comes as close as possible to achieving the target population RDA for all nutrients, while adhering to dietary patterns (median food group consumption) as much as possible. These diets minimize the sum of the deviations from the RDA for all nutrients, and minimize deviations from food patterns. **Optimal diet, without food patterns:** Diet that comes as close as possible to achieving the target population RDA for all nutrients, not accounting for dietary patterns. These diets minimize the sum of the deviations from the RDA for all nutrients (but are only subject to maximum and minimum food group constraints). Large differences in % RDA between the two diets can be used to identify food groups and food sub-groups which changed substantially between the two diets, and can be used to generate food based recommendations. [3]Energy constraints, food serving sizes and food consumption patterns are presented in Tables 1 and 4, and Supplemental Tables 1-3. [4]Series modeled using calcium RDA of 1000 mg/d.

Table S7. The nutrient content of optimal diets with and without food patterns (module II), expressed as a percentage of Recommended Dietary Allowances (RDA) among lactating women[1,2.]

**Analysis**[3]	**% of RDA**										
	Vitamin A	Vitamin C	Thiamin	Riboflavin	Niacin	Vitamin B_6	Folate	Vitamin B_{12}	Iron	Zinc	Calcium[4]
**Reported energy intake**											
Optimal diet, with food patterns	33.6	24.8	77.1	66.3	51	113.7	51.7	4.3	206.8	156.5	32.7
Optimal diet, without food patterns	49.2	40.8	87.1	66.5	66.5	95.2	78.9	21.2	151.2	156.8	50.4
**Added meal**											

Optimal diet, with food patterns	45.3	39.6	89.8	91.1	60.5	132.6	64.3	4.5	249.9	194.4	45.9
Optimal diet, without food patterns	80.1	67.2	100.0	100.0	80.8	105.3	100.0	39.4	181.9	212.6	85.4
**Added meal + UNIMMAP**											
Optimal diet, with food patterns	105.2	97.7	189.1	176.0	166.2	230.6	199.9	97.6	418.2	320.1	49.1
Optimal diet, without food patterns	126.2	113.5	196.5	194.5	183.4	220.1	240.9	132.4	423.4	365.4	88.2
**Added meal + Supercereal (CSB+)**											
Optimal diet, with food patterns	119.0	100.1	116.8	177.2	112.0	187.9	89.4	75.7	282.2	233.2	99.0
Optimal diet, without food patterns	129.5	99.0	132.3	166.6	125.0	179.5	100.7	99.0	170.1	252.7	107.8
**Added meal + SQ-LNS P & L**											
Optimal diet, with food patterns	105.5	121.7	284.4	261.1	269.6	321.0	197.5	190.5	352.1	436.9	77.8
Optimal diet, without food patterns	99.0	112.5	309.0	250.6	292.2	315.7	238.1	215.4	249.4	451.8	99.0
**Added meal + Plumpy'Mum**											
Optimal diet, with food patterns	157.6	102.8	185.9	157.4	168.9	217.7	169.3	102.8	190.6	268.3	47.1
Optimal diet, without food patterns	164.3	131.3	197.2	206.6	177.1	221.8	234.9	144.1	262.3	365.1	96.7

[1] CSB+, corn soy blend plus; IFA, iron and folic acid supplement; SQ-LNS, small-quantity lipid-based nutrient supplement; P&L, pregnancy and lactation. [2] **Optimal diet, with food patterns**: Diet that comes as close as possible to achieving the target population RDA for all nutrients, while adhering to dietary patterns (median food group consumption) as much as possible. These diets minimize the sum of the deviations from the RDA for all nutrients, and minimize deviations from food patterns. **Optimal diet, without food patterns:** Diet that comes as close as possible to achieving the target population RDA for all nutrients, not accounting for dietary patterns. These diets minimize the sum of the deviations from the RDA for all nutrients (but are only subject to maximum and minimum food group constraints). Large differences in % RDA between the two diets can be used to identify food groups and food sub-groups which changed substantially between the two diets, and can be used to generate food based

recommendations. [3] Energy constraints, food serving sizes and food consumption patterns are presented in Tables 1 and 4, and Supplemental Tables 1-3. [4] Series modeled using calcium RDA of 1000 mg/d.

Table S8. The nutrient content of worst-case and best-case scenario diets without food-based recommendations (module III), and food-based recommendations with the greatest nutritional impact expressed as a percentage of Recommended Dietary Allowances (RDA) among pregnant and lactating women [1,2].

	% of RDA												
**Analysis** [3]	Vitamin A	Vitamin C	Thiamin	Riboflavin	Niacin	Vitamin B_6	Folate	Vitamin B_{12}	Iron	Zinc	Calcium [4]	No. MN Adequate	Cost of Diet (€/d)
**Pregnant Women**													
**Added meal + 1 egg/d**													
Best modeled FBR (worst-case scenario)	86.9	26.3	79.4	103.4	51.1	109.5	78.3	67.0	109.0	213.1	69.6	9	0.60
**Added meal + 100 g red meat/d**													
Best modeled FBR (worst-case scenario)	74.5	26.3	81.6	97.0	84.3	109.8	76.3	138.4	112.3	245.1	68.0	10	0.68
**Lactating Women**													
**Added meal + 1 egg/d**													
Best modeled FBR (worst-case scenario)	21.1	15.1	80.3	89.0	55.2	114.3	75.3	62.3	185.3	210.3	68.6	7	0.60
**Added meal + 100 g red meat/d**													
Best modeled FBR (worst-case scenario)	13.7	15.1	83.2	83.9	91.6	117.0	73.1	128.5	190.8	241.8	67.0	9	0.68

[1]RDA, recommended dietary allowances. [2] Worst-case scenario: diets sequentially modeled for each micronutrient, which would provide the least possible amount (expressed as % of the RDA) of that micronutrient. Dietary adequacy for each nutrient was defined as the worst-case scenario for that nutrient being ≥ 65% of the RDA (shaded); if the worst-case scenario is less than 65% (non-shaded) of the RDA, the nutrient is likely to be inadequate in the population, given local food supply and food patterns. [3] Energy constraints, food serving sizes and food consumption patterns are presented in Tables 1 and 4, and Supplemental Tables 1-3. Best-modeled FBR for

pregnant women included (per week): 7 eggs or 700 g red meat, 21 servings of dark green leafy vegetables, 14 servings of milk, 14 servings of millet, 21 servings of vitamin A fortified vegetable oil and 21 servings of cooked beans/lentils/peas. Best-modeled FBR for lactating women included (per week): 7 eggs or 700 g red meat, 21 servings of dark green leafy vegetables, 14 servings of milk, 14 servings of millet and 21 servings of cooked beans/lentils/peas. [4] Series modeled using calcium RDA of 1000 mg/d.

Table S9. The nutrient content of worst-case and best-case scenario diets without food-based recommendations (module III), and food-based recommendations with the greatest nutritional impact expressed as a percentage of Recommended Dietary Allowances (RDA) among pregnant women, using lower calcium recommendations (775 mg/d) [1,2].

	% of RDA												
**Analysis** [3]	**Vitamin A**	**Vitamin C**	**Thiamin**	**Riboflavin**	**Niacin**	**Vitamin B_6**	**Folate**	**Vitamin B_{12}**	**Iron**	**Zinc**	**Calcium** [4]	**No. MN Adequate**	**Cost of Diet (€/d)**
Added meal + UNIMMAP													
Best modeled FBR (worst-case scenario)	134.0	107.8	152.9	160.0	151.4	184.5	128.3	139.2	152.4	302.7	75.5	11	0.36
Added meal + Supercereal (CSB+)													
Best modeled FBR (worst-case scenario)	142.6	86.4	85.5	133.7	86.6	117.3	69.0	79.2	73.6	145.1	69.9	11	0.19
Added meal + SQ-LNS P & L													
Best modeled FBR (worst-case scenario)	122.1	140.5	252.4	240.7	242.9	284.4	127.4	202.1	115.4	373.0	72.6	11	0.22
Added meal + Plumpy'Mum													
Best modeled FBR (worst-case scenario)	138.5	107.7	146.0	154.7	153.6	166.5	141.5	119.3	158.2	253.1	68.8	11	0.23

[1] CSB+, corn soy blend plus; SQ-LNS, small-quantity lipid-based nutrient supplement; P&L, pregnancy and lactation. [2]Worst-case scenario: diets sequentially modeled for each micronutrient, which would provide the least possible amount (expressed as % of the RDA) of that micronutrient. Dietary adequacy for each nutrient was defined as the worst-case scenario for that nutrient being ≥ 65% of the RDA (shaded); if the worst-case scenario is less than 65% (non-shaded) of the RDA, the nutrient is likely to be

inadequate in the population, given local food supply and food patterns. [3] Energy constraints, food serving sizes and food consumption patterns are presented in Tables 1 and 4, and Supplemental Tables 1-3. Best-modeled FBR are presented in Table S11 for each series. [4] Series modeled using calcium RDA of 775 mg/d.

Table S10. The nutrient content of worst-case and best-case scenario diets without food-based recommendations (module III), and food-based recommendations with the greatest nutritional impact expressed as a percentage of Recommended Dietary Allowances (RDA) among lactating women, using lower calcium recommendations (800 mg/d) [1,2].

	% of RDA												
**Analysis [3]**	**Vitamin A**	**Vitamin C**	**Thiamin**	**Riboflavin**	**Niacin**	**Vitamin B_6**	**Folate**	**Vitamin B_{12}**	**Iron**	**Zinc**	**Calcium [4]**	**No. MN Adequate**	**Cost of Diet (€/d)**
Added meal + UNIMMAP													
Best modeled FBR (worst-case scenario)	75.1	72.9	156.2	135.9	161.0	175.1	152.0	129.4	234.7	286.0	71.8	11	0.38
Added meal + Supercereal (CSB+)													
Best modeled FBR (worst-case scenario)	88.9	69.7	91.3	123.6	97.1	129.1	74.6	73.6	125.2	143.3	87.1	11	0.21
Added meal + SQ-LNS P & L													
Best modeled FBR (worst-case scenario)	68.0	96.1	250.8	204.9	255.4	265.1	149.6	187.7	174.9	341.4	68.6	11	0.23
Added meal + Plumpy'Mum													
Best modeled FBR (worst-case scenario)	77.8	72.8	152.0	131.2	164.0	162.4	168.7	110.9	243.4	242.2	65.2	11	0.25

[1] CSB+, corn soy blend plus; SQ-LNS, small-quantity lipid-based nutrient supplement; P&L, pregnancy and lactation. [2]Worst-case scenario: diets sequentially modeled for each micronutrient, which would provide the least possible amount (expressed as % of the RDA) of that micronutrient. Dietary adequacy for each nutrient was defined as the worst-case scenario for that nutrient being ≥ 65% of the RDA (shaded); if the worst-case scenario is less than 65% (non-shaded) of the RDA, the nutrient is likely to be inadequate in the population, given local food supply and food patterns. [3] Energy constraints, food serving sizes and food consumption patterns are presented in Tables 1 and 4, and Supplemental Tables 1-3. Best-modeled FBR are presented in Table S11 for each series. [4] Series modeled using calcium RDA of 800 mg/d.

Table S11. Food-based recommendations for pregnant and lactating women using lower calcium recommendations (775-800 mg/d), by model series and intervention product [1].

	Pregnant Women	No. MN Adequate [2]	Lactating Women	No. MN Adequate
Added meal diet + UNIMMAP	• 21 servings of DGLV<br>• 14 servings of milk	11	• 21 servings of DGLV<br>• 14 servings of milk	11
Added meal diet + Supercereal (CSB+)	• 14 servings of cooked beans/lentils/peas	11	• 14 servings of DGLV<br>• 14 servings of cooked beans/lentils/peas	11
Added meal + SQ-LNS (P&L)	• 21 servings of DGLV	11	• 21 servings of DGLV	11
Added meal diet + Plumpy'Mum	• 21 servings of DGLV<br>• 7 servings of milk	11	• 21 servings of DGLV<br>• 7 servings of milk	11

[1] CSB+, corn soy blend plus; DGLV, dark green leafy vegetables; MN, micronutrients; SQ-LNS, small-quantity lipid-based nutrient supplement; P&L, pregnancy and lactation. [2] Maximum number of micronutrients with the potential to be adequate, $n = 11$. [3] Added meal diet: model energy constraint increased above the reported diet to approximate the provision of an "added meal" (~600 kcal) per day among pregnant women or to match estimated energy requirements in lactating women, in addition to the best set of food-based recommendations.
